# Optimal risk assessment intervals for primary prevention of cardiovascular disease: a population-based two-stage landmarking study

**DOI:** 10.1136/bmjph-2024-001241

**Published:** 2025-03-28

**Authors:** Zander Gu, Francesca Gasperoni, Ellie Paige, Michael Sweeting, Juliet Usher-Smith, Katrina Poppe, David Stevens, Matthew Arnold, Emanuele Di Angelantonio, Angela M Wood, Jessica K Barrett

**Affiliations:** 1 MRC Biostatistics Unit, University of Cambridge, Cambridge, UK; 2 Population Health Program, QIMR Berghofer Medical Research Institute, Brisbane, Queensland, Australia; 3 School of Public Health, The University of Queensland, Brisbane, Queensland, Australia; 4 National Centre for Epidemiology and Population Health, Australian National University, Canberra, Australian Capital Territory, Australia; 5 University of Leicester, Leicester, UK; 6 Primary Care Unit, Department of Public Health and Primary Care, University of Cambridge, Cambridge, UK; 7 Department of Medicine, University of Auckland, Auckland, New Zealand; 8 British Heart Foundation Cardiovascular Epidemiology Unit, Department of Public Health and Primary Care, University of Cambridge, Cambridge, UK; 9 Victor Phillip Dahdaleh Heart and Lung Research Institute, University of Cambridge, Cambridge, UK; 10 British Heart Foundation Centre of Research Excellence, University of Cambridge, Cambridge, UK; 11 National Institute for Health and Care Research Blood and Transplant Research Unit in Donor Health and Behaviour, University of Cambridge, Cambridge, UK; 12 Health Data Research UK Cambridge, Wellcome Genome Campus and University of Cambridge, Cambridge, UK; 13 Health Data Science Research Centre, Human Technopole, Milan, Italy

**Keywords:** Public Health, Cardiovascular Diseases, Risk Assessment, Epidemiologic Methods, Primary Prevention

## Abstract

**Introduction:**

The recommended assessment intervals for primary prevention of cardiovascular disease (CVD) differ in major international guidelines. We aimed to provide empirical evidence on the optimal frequency of CVD risk assessment to inform future guidelines.

**Methods:**

We estimated the expected time to cross the 10-year CVD risk treatment threshold of 10% using extended two-stage landmarking for more than 2 million people using UK primary care electronic health records between April 2004 and May 2019 from the Clinical Practice Research Datalink GOLD Database (CPRD GOLD), which was linked to hospital admissions data from the Hospital Episodes Statistics (HES) dataset and national mortality records from the Office for National Statistics (ONS). We grouped people based on their sex, initial risk level and age, and computed various percentiles of the expected crossing times per group. Based on the percentiles, optimal assessment intervals were identified and their performance was evaluated comparing to the current recommended intervals in the UK.

**Results:**

Our results showed that the expected crossing times for people with lower initial risk were much longer than those with higher initial risk. Within each initial risk group, expected time to crossing the risk treatment thresholds was shorter in people aged ≥65 years. Based on the median expected crossing times, our recommended intervals for women with initial 10-year risk of 7.5%–10%, 5%–7.5%, 2.5%–5% or<2.5% are 3 (1 if ≥65 years old), 7 (4), 10 (6) and 10 (10) years, respectively; intervals for men are 2 (1), 5 (5), 9 (9) and 10 (10) years. These intervals outperformed the 5-yearly risk reassessment for all individuals currently recommended in the UK.

**Conclusions:**

Our evidence suggests that CVD risk assessment intervals for primary prevention should be stratified by sex, initial risk level and age. For the UK population, our method found risk assessment intervals that reduce the number of assessments required while shortening the waiting time to the next assessment for those most in need.

**WHAT IS ALREADY KNOWN ON THIS TOPIC**

- Identifying optimal intervals for reassessing cardiovascular disease (CVD) risk is important for ensuring preventive treatments can be started as early as possible for people at high risk. To date, only two studies published empirical data on the intervals for assessing CVD risk. Both studies concluded that risk assessment intervals should be based on initial estimates of risk. However, sample sizes were small and measurements were only available at a few specified time points, often outside the intervals considered in clinical practice guidelines and with no data in between.

**WHAT THIS STUDY ADDS**

- This is the first large-scale study providing empirical evidence on the optimal times to reassess CVD risk, using UK primary care data from more than 2 million people. Our study adds to the previous literature by providing the distributions of the expected time to cross a given CVD risk treatment threshold for subpopulations stratified by sex, age and initial risk level. Based on these expected times, we recommend sex-specific, risk-specific and age-stratified assessment intervals for the UK context, which outperform the current recommendation of 5-yearly risk reassessment for all individuals. Our results are also relevant beyond the UK and we provide the methodological framework to derive the optimal assessment intervals that can be readily applied to primary care data from other regions.

**HOW THIS STUDY MIGHT AFFECT RESEARCH, PRACTICE OR POLICY**

- Our study suggests that guideline recommendations for CVD risk assessment intervals should be stratified based on initial level of risk, age and sex. While more complex to apply than blanket approaches such as 5-yearly risk assessment, stratification by risk, age and sex can reduce the number of risk assessments needed and ensure treatments are targeted as early as possible to those with greatest need. Further research on the implications on costs and health outcomes is needed.

## Introduction

 Cardiovascular disease (CVD) is a leading cause of death and disability globally yet is highly preventable.^1^ The risk of a first CVD event can be reduced through lifestyle-related changes and the use of lipid-lowering and blood pressure-lowering medications which each reduce the risk of a future event by around 20–25%.^2 3^ Assessing a person’s risk of having a CVD event using a validated risk equation and implementing preventative measures according to this risk is the cornerstone of CVD primary prevention. In major international guidelines for CVD prevention, there are differences in both the risk thresholds recommended for treatment and in the recommended frequency for assessing risk. Guideline recommendations are typically based on a combination of evidence, where it exists, and input from clinicians, other experts and patients’ preferences and expectations, which can differ between regions.

Risk treatment thresholds vary between guidelines from ≥5% over 10 years in Norway (for people aged 45–54 years) to >20% over 10 years for WHO recommendations.^4^,^6^ Within Europe, lipid-lowering therapy is recommended for those with a ≥10% 10-year risk for people aged 40–84 years in the UK and those aged 50–69 years in other European countries.^7 8^ The time intervals between risk assessments are not always explicitly stated in guidelines, and where they are, they are generally either recommended for a specified time period (eg, 5 years for the UK, Europe, Canada) or vary depending on a person’s initial estimated risk level (eg, Australia, New Zealand).^9^,^11^

Ideally, CVD risk should be assessed frequently enough that those who have crossed the risk treatment threshold are identified within a reasonable timeframe so that preventative treatments can be discussed as part of a shared decision-making process. However, there are little empirical data to guide decisions around optimal risk assessment intervals, and this is likely to differ based on age and how close someone is to an existing risk treatment threshold, that is, their initial risk level. Only two articles have published empirical data on the optimal frequency for assessing risk, both finding that CVD risk assessment intervals are more optimal when based on initial risk level. In one study of 17 612 participants from the Tokyo health check-up and Framingham heart study, the probability of crossing a 20% risk treatment threshold at seven specified time periods (1, 2, 3, 8, 12, 16 and 19 years) was examined.^12^ The probability of crossing the treatment threshold varied by initial risk but no data were available for 4 to 7 years, the time points most commonly recommended in guidelines for reassessing risk. This was particularly problematic for those at initial moderate risk, where substantial proportions of people crossed the risk treatment threshold between 3 and 8 years (eg, among those with an initial 10%–15% 10-year risk, 5.7% of individuals had a risk above the 20% risk treatment threshold at 3 years and 32.1% were above the threshold by 8 years).

Lindbohm and colleagues expanded on this,^13^ using 5-yearly follow-up data from 6964 participants in the British Whitehall II study to compare 5-yearly risk reassessment to 21 different risk assessment schedules that varied based on initial level of risk. They found that intervals for CVD risk assessment that varied based on initial risk level resulted in less time spent at risk without assessment, fewer CVD events and reduced QALYs and costs compared with risk assessment based on a blanket 5-year reassessment. However, risk assessment was measured only four times over an average of 22 years of follow-up time. Data between follow-up times were modelled using a multistate model; transitions between risk categories were modelled under the strong assumption of proportional hazards with a constant baseline risk, which may have decreased accuracy in the projected risk estimations. Furthermore, no data were available on the average time to cross a given risk treatment threshold within specific risk categories.

Our study aimed to provide empirical evidence on the optimal times to reassess CVD risk, using large-scale UK primary care data from more than 2 million people. We quantified the expected time to cross a given CVD risk treatment threshold, by sex, age and initial risk level, taking advantage of repeated measurements of CVD risk factors over time. The findings are used to provide evidence-based recommendations on the optimal frequency of CVD risk assessment to inform guidelines for primary prevention of CVD.

## Methods

### Study design and population

We analysed UK primary care electronic health records from the Clinical Practice Research Datalink GOLD Database (CPRD GOLD), linked to hospital admissions data from the Hospital Episodes Statistics (HES) dataset and national mortality records from the Office for National Statistics (ONS).^14^ The dataset extracted by CPRD from the CPRD GOLD database contains anonymised primary care records of 17 251 881 individuals and is broadly representative in terms of age, sex and ethnicity.^14^

Eligible participants entered the study from the latest of the date 6 months after registration at the general practice; the date the individual turned 40 years of age (note, prior information from age 30 years onward was extracted for these individuals); the date that the data for the practice were assessed as being up to standard^15^; or 1 April 2004, the date of introduction of the Quality and Outcomes Framework.^16^ Participants were followed up until date of death or first CVD diagnosis; the date that the individual turned 95 years of age; the date that last data were available for the individual in CPRD; or 31 May 2019 (end date of data availability across the study), whichever came first.

Of the 2 525 156 individuals with linked data, those with a prior diagnosis of CVD or statin prescription before study entry were excluded. We only included individuals who had data on at least one of body mass index (BMI), systolic blood pressure (SBP), total cholesterol, high-density lipoprotein (HDL) cholesterol and smoking status during the study period.

### Primary outcome

The main outcome of interest was the first non-fatal or fatal CVD event recorded in primary care, hospital admissions or cause of death datasets. CVD was defined according to the outcome coding used in the QRISK algorithm,^17^,^19^ which is recommended for use in the UK and included coronary heart disease (including myocardial infarction and angina), stroke and transient ischaemic attack (code list in online supplemental table S1.^7^

### CVD risk predictors

We quantified CVD risk using risk factors included in the QRISK2/3 equations.^18 19^ Specifically, we included BMI, SBP, total cholesterol, HDL cholesterol, smoking status, level of deprivation measured using the quintiles of the Townsend score,^20^ blood pressure-lowering medication prescription, and previous diagnoses of diabetes, renal disease, migraine, rheumatoid arthritis, depression, severe mental illness and atrial fibrillation. Code lists for disease diagnoses and biologically implausible risk factor ranges are outlined in the online supplemental material. Individuals with a specific disease diagnosis or prescription were assumed to have the condition for the rest of follow-up after their first prescription or diagnosis.

### Statistical model

Due to restrictions of the maximum follow-up, analysis was conducted to determine 5-year CVD risks which were then transformed to 10-year risks using sex-stratified, age-stratified and risk-stratified models (details provided below). The 5-year CVD risks were predicted using a dynamic prediction model built using two-stage landmarking and the extension of Gasperoni *et al* to evaluate future CVD risks.^21^,^23^ The landmark model is a statistically robust approach for analysing electronic health records, allowing repeated measurements of risk factors to be included in the analysis while accounting for measurement error and unobserved data which can be common in electronic health records.

#### Landmark cohort

A landmark cohort for each 5-year age group (40, 45,…, 80, denoted as “landmark ages”) was specified as individuals who (1) were in the study at the landmark age; (2) did not have a CVD event prior to the landmark age; (3) had at least one measurement of one of BMI, SBP, total cholesterol, HDL cholesterol and smoking status; and (4) had no statin prescription prior to the landmark age.

#### Two-stage landmarking

In stage one, trajectories for risk factors BMI, SBP, total cholesterol, HDL cholesterol and smoking status were modelled for each landmark age using a sex-specific, multivariate, linear mixed effects model with random intercepts and random slopes of age. Values of BMI, SBP, total cholesterol and HDL cholesterol were standardised using sex-specific means and SD. For SBP and total cholesterol, we included blood pressure-lowering medication and statin prescription (initiated after the landmark age) as covariates, respectively.

In stage two, at each landmark age, we fitted two sex-specific Cox proportional hazards models for the time from the landmark age to the time of first CVD event: one model used event-time data censored at 5 years and the other at 10 years. The 10-year Cox models were used to (1) predict 10-year risk at the landmark age and categorise people into five groups based on the predicted risk: ≥10%, 7.5%–10%, 5%–7.5%, 2.5%–5% and <2.5%; and (2) find the correspondence between 10-year and 5-year risks (see the next paragraph). Risk predictors included in the Cox model were current values of BMI, SBP, total cholesterol, HDL cholesterol and smoking status (predicted using the mixed effects model fitted in stage one), and all other QRISK 2/3 risk predictors specified earlier.

#### 10-year to 5-year risk ratios

We computed the ratios of the 10-year risk to the 5-year risk stratified by sex, landmark age and initial risk level (details in online supplemental material). The risk ratios were then used to convert the predicted 5-year risks to 10-year risks (see the next paragraph). We used 5-year risk to approximate 10-year risk because the maximum follow-up time was 15 years, which was only sufficient to predict 5-year risk at all times of interest (0–10 years after the landmark age).

#### Times to crossing the risk treatment threshold

**Figure 1:**
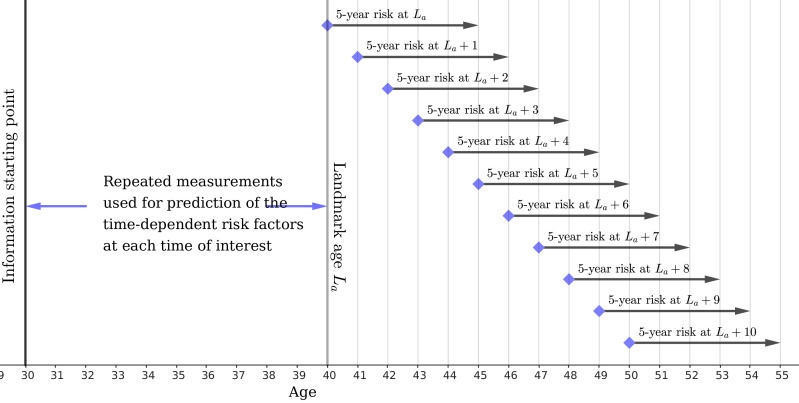
Illustration of the extended two-stage landmarking model for $L_{a}$=40. Note that in the first stage, all the measurements (including the ones after the landmark age) were used for fitting LMEM; in the second stage, only the measurements before the landmark age were used for prediction.

At each future ‘time of interest’, considered to be each year after the landmark age up to 10 years, a Cox model was fitted for the time from the ‘time of interest’ to the first CVD event (censored at 5 years) for all individuals still at risk of a primary CVD event. See figure 1 for an illustration. The predicted 5-year risks were then converted to 10-year risks by multiplying the corresponding 10-year to 5-year risk ratio. Risk predictors were predicted values at the ‘time of interest’ of BMI, SBP, total cholesterol, HDL cholesterol and smoking status, and other CVD risk predictors specified earlier.

The expected time of crossing the 10-year risk of 10% (which is recommended in the UK^7^) was linearly interpolated between the first ‘time of interest’ when the 10-year CVD risk exceeds the threshold, and the previous ‘time of interest’ (up to 10 years or >10 years if the risk threshold of 10% is not crossed).

### Percentiles of expected crossing times

We categorised people in each landmark cohort based on their 10-year risk predicted at landmark age: ≥10%, 7.5%–10%, 5%–7.5%, 2.5%–5% and <2.5%. People in the ≥10% group were excluded from the calculation of expected crossing times as their initial risk puts them above the risk threshold for initiating pharmacotherapy. For each of the remaining risk groups, we computed the 50th, 25th and 10th percentiles (denoted P50, P25 and P10, respectively) of the expected crossing times per landmark age (note, the 50th percentile represents the median expected time to cross the risk treatment threshold for the participants in that specific age and risk group). The average expected crossing time per risk group was then calculated as an average weighted by 2020 UK population demographics (details in online supplemental material). Age-stratified average crossing times were also calculated for landmark age groups of <65 and ≥65 years within each risk group.

### Assessment strategies

We identified one risk-specific and one risk-specific and age-stratified (<65 and ≥65) assessment strategy for each sex using each of the 50th, 25th and 10th percentiles of crossing times. The assessment interval for a specific risk and age (for age-stratified) group was rounded up to the nearest year (or rounded down if the first decimal place is 0), or 10 years if the average percentile was above 10 years.

To compare the recommended strategies to the current recommended approach of assessing risk every 5 years in the UK, we computed metrics for the expected number of follow-up assessments per person within 10 years, the proportion crossing the risk treatment threshold before first risk assessment, and the average waiting time to the first follow-up risk assessment after crossing the risk threshold.

See the Supplementary material for more details of all statistical methods.

## Results

### Cohort characteristics

A total of 2 080 115 individuals (1 081 340 women and 998 775 men) were included in the study (online supplemental figure S1).

The characteristics of participants are listed in table 1. At study entry, the mean age was 51.0 (SD, 13.0) years, and 48% of the participants were men. The median follow-up was 8.1 years (IQR, 3.9–11.3), during which 133 038 (6.4%) individuals had a CVD event, with a higher proportion of men experiencing a CVD event than women (7.4% vs 5.4%).

**Table 1:** Characteristics of participants

	Women		Men	
	Mean (SD)/cases (%)	Sample size*	Mean (SD)/cases (%)	Sample size*
Sample size N	1 081 340		998 775	
CVD event†	58 834 (5.4%)	1 081 340	74 204 (7.4%)	998 775
Age at study entry, years	51.7 (13.5)	1 081 340	50.2 (12.5)	998 775
Time-dependent				
Body mass index‡, kg/m^2^	27.4 (6.3)	739 373	27.8 (5.2)	575 575
Systolic blood pressure‡, mm Hg	131.0 (19.0)	921 391	136.0 (17.2)	758 826
Total cholesterol‡, mmol/L	5.5 (1.1)	658 018	5.3 (1.1)	577 955
HDL cholesterol‡, mmol/L	1.6 (0.4)	604 664	1.3 (0.4)	531 727
Current/ever smoker‡	168 225 (37.7%)	445 977	189 738 (40.1%)	473 419
Time-fixed				
Townsend§ 1/2/3/4/5	25/24/21/18/12 %	1 076 950	24/23/21/19/13 %	994 114
BP-lowering medication¶	395 794 (36.6%)	1 081 340	313 134 (31.4%)	998 775
Diabetes¶	52 943 (4.9%)	1 081 340	82 964 (8.3%)	998 775
Renal disease¶	79 664 (7.4%)	1 081 340	52 265 (6.1%)	998 775
Migraine¶	121 343 (11.2%)	1 081 340	36 582 (4.4%)	998 775
Rheumatoid arthritis¶	22 567 (2.1%)	1 081 340	8626 (1.0%)	998 775
Depression¶	315 049 (29.1%)	1 081 340	146 636 (17.6%)	998 775
Severe mental illness¶	19 967 (1.8%)	1 081 340	15 545 (1.9%)	998 775
Atrial fibrillation¶	30 667 (2.8%)	1 081 340	34 259 (4.1%)	998 775

* Number of individuals with at least one measurement.

† Number (%) of individuals who experienced CVD event during the follow-up.

‡ Value of the first observed measurement after study entry.

§ Quintiles of the Townsend score measuring the level of deprivation, 1 is least deprived and 5 is most deprived.

¶ Number(%) of individuals who are prescribed medication or diagnosed during the follow-up at any point.

Most individuals had all of the time-dependent risk factors (BMI, SBP, total cholesterol, HDL cholesterol, smoking status) measured (online supplemental figure S2). For each risk factor, a histogram showing the number of measurements per individual is shown in online supplemental figure S3.

### Estimates from the landmark models

Regression coefficients from multivariate linear mixed effects models and hazard ratios for the Cox models (censored at 5 years after each landmark age) are provided in online supplemental figures S4-S7. Overall, the fixed intercepts from the multivariate mixed effects models show that average HDL cholesterol and SBP increased over the landmark ages, whereas smoking prevalence decreased. The average BMI and total cholesterol increased over landmark ages in younger ages and decreased in older ages (online supplemental figure S4). Among these time-dependent factors, higher values of SBP, total cholesterol and higher prevalence of smoking were associated with increased hazards for CVD, especially below age 70 years. In contrast, higher HDL cholesterol was associated with decreased hazard ratios for CVD. Hazard ratios for BMI were close to the null at all ages (online supplemental figure S5).

### 10-year to 5-year risk ratios

**Figure 2:**
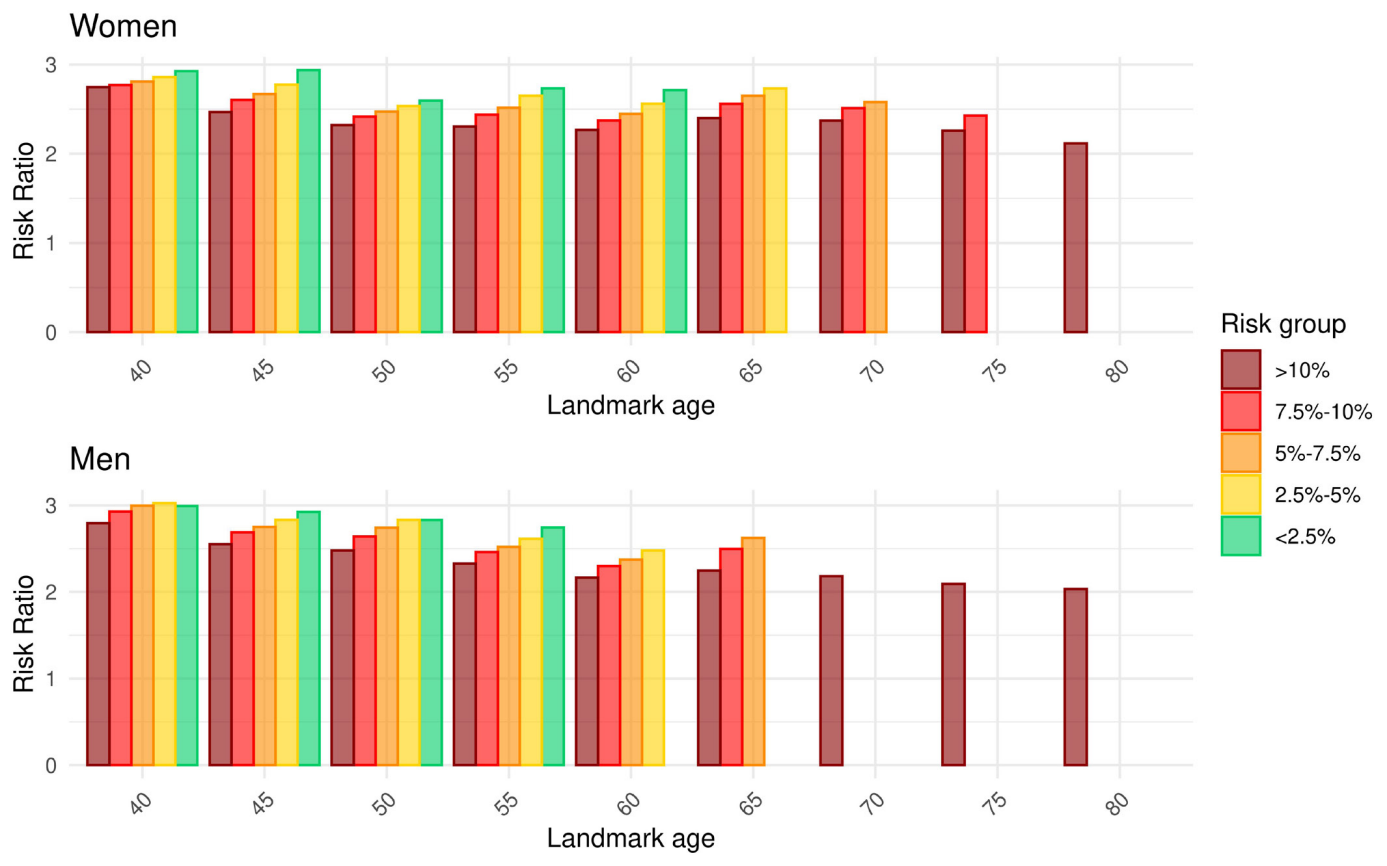
Ratios of 10-year risk to 5-year risk predicted at each landmark age stratified by initial 10-year risk level for women (above) and men (below). The missing bars indicate empty risk groups under each landmark cohort.

The risk ratios between 10-year and 5-year risk stratified by sex, landmark age and initial risk level are shown in figure 2. Overall, the ratios decreased from approximately 3 in younger landmark cohorts to approximately 2 in older landmark cohorts for both sexes. Within each landmark cohort, the ratio for people with lower initial risk level tended to be higher.

### Distributions of the expected crossing times

The proportions of individuals who had an initial 10-year risk above 10% are given in the online supplemental table S2, with the proportion increasing sharply with age. By the age of 75 years in women and 65 in men, almost everyone had an initial estimated 10-year CVD risk greater than the 10% risk treatment threshold. Men were more likely than women to have crossed the risk treatment threshold at each landmark age.

**Figure 3:**
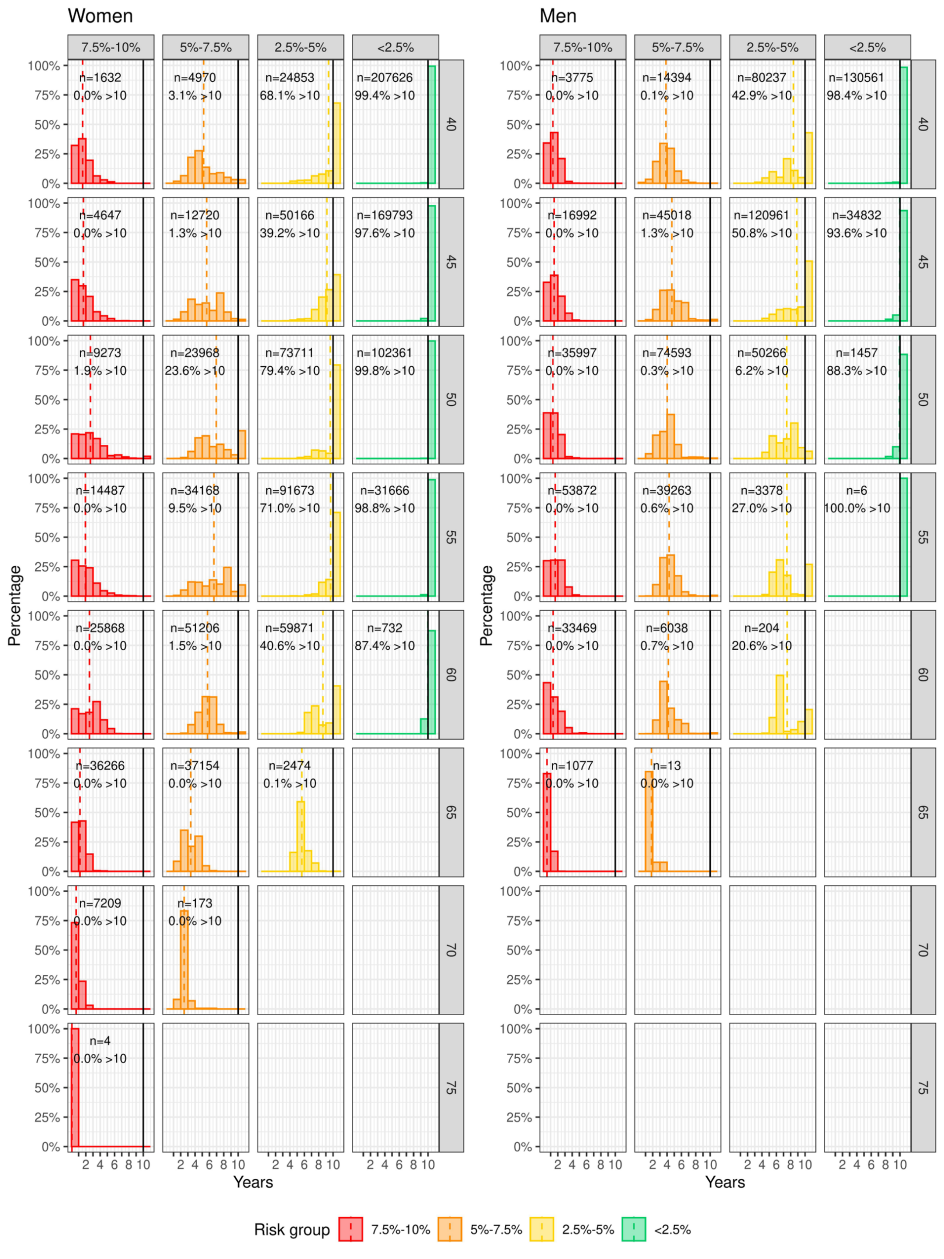
Distribution of expected crossing times for women (left panel) and men (right panel). Columns are risk groups and rows are landmark ages. The dotted vertical lines indicate the mean (the expected crossing times of those whose crossing time was greater than 10 years were set to 10 years for computing the mean). The solid vertical lines indicate the maximum of 10 years. The bar to the right of the solid vertical line shows the proportion of individuals who did not cross the threshold in 10 years. The total number of individuals and the percentage of individuals who did not cross the threshold in each subgroup are shown in the corresponding subplot. Graphs are not shown for empty groups. Note that the groups where n<100 were removed from subsequent analyses.

The distribution of the expected crossing times by risk group and landmark age is shown in figure 3. Overall, people with a higher estimated risk level and older people tended to have a shorter expected crossing time. Most individuals (with a minimum proportion of 87.4%) with an initial estimated 10-year risk of <2.5% did not cross the threshold within 10 years, regardless of age and sex. These observations were confirmed by the crossing time percentiles stratified by risk group and by risk group and age, shown in online supplemental figure S8. The percentiles of the crossing times for those with a higher initial CVD risk were substantially smaller than those with a lower initial CVD risk, and the percentiles for the subcohorts aged ≥65 years were lower than those for the subcohorts aged <65 years. The percentiles of crossing times per risk group per landmark age for each sex are listed in online supplemental table S3.

### Comparison of risk assessment strategies

The risk-specific age-stratified and non-age-stratified assessment strategies for each sex using the crossing time percentile as the recommended reassessment interval are listed in table 2, together with the benchmark strategy of reassessment every 5 years for comparison. The expected number of follow-up assessments per person within 10 years, proportion crossing the threshold before the first risk assessment, and the average waiting time to the first follow-up risk assessment after crossing the risk threshold for each sex and the whole population are also listed for each strategy.

**Table 2:** Summary of the risk assessment intervals for strategies based on various percentiles of expected crossing time

	Benchmark	P50*		P25		P10	
		Non-stratified	Age-stratified	Non-stratified	Age-stratified	Non-stratified	Age-stratified
Women							
Intervals†	5-5-5-5	2-6-10-10	<65: 3-7-10-10≥65: 1-4-6-/	1-5-9-10	<65: 1-5-9-10≥65: 1-3-6-/	1-4-8-10	<65: 1-4-8-10≥65: 1-2-5-/
No. assessments	2	1.56	1.75	2.21	2.28	2.34	2.45
% crossed	19	30.1	30.2	20.3	18	13.9	11.2
Wait time‡	2.54	1.65	1.59	1.22	1	0.89	0.73
Men							
Intervals	5-5-5-5	2-5-9-10	<65: 2-5-9-10≥65: 1-/-/-/	1-4-8-10	<65: 1-4-8-10≥65: 1-/-/-/	1-3-6-10	<65: 1-3-6-10≥65: 1-/-/-/
No. assessments	2	2.22	2.24	3.56	3.56	3.91	3.91
% crossed	44.4	53.2	53.2	32.5	32.5	17.7	17.7
Wait time	2.45	1.44	1.43	1.04	1.04	0.59	0.59
All							
No. assessments	2	1.83	1.94	2.75	2.79	2.97	3.03
% crossed	29.1	39.3	39.4	25.2	23.8	15.4	13.8
Wait time	2.49	1.54	1.51	1.13	1.02	0.75	0.66

* P50 represents the median time to crossing the 10-year risk treatment threshold of 10%.

† Reassessment interval in years for the risk group of 7.5%–10%, 5%–7.5%, 2.5%–5% and <2.5%, respectively, for example, 5-5-5-5 signifies a strategy of 5-yearly assessment intervals for all initial risk groups. ‘/’ indicates no data available due to empty intersection of lower initial risk and older age groups.

‡ Wait time is the average number of years until next risk assessment after crossing the 10-year risk treatment threshold of 10%.

Comparing the P50 strategies based on the median time to crossing the 10-year risk treatment threshold of 10% to the benchmark approach of assessing risk every 5 years, the expected number of follow-up assessments per person was reduced from 2 to 1.56 across all ages and to 1.75 for the age-stratified approach for women (table 2). The number of risk assessments was slightly increased in men, to 2.22 across all ages and 2.24 for the age-stratified approach. Combining women and men, the number of assessments decreased from 2 to 1.83 (non-stratified) and 1.94 (age-stratified). At the time of the first risk assessment, the proportion of people who had crossed the risk threshold increased by 30% in the whole population from 29.1% (benchmark) to around 39% (P50 strategies). The waiting time to risk reassessment after crossing the threshold was reduced for both men and women in both non-stratified and age-stratified P50 strategies compared with the benchmark 5-year strategy. For the whole population, the average waiting time was reduced by approximately 40% from 2.49 years to 1.54 years (non-stratified) and 1.51 years (age-stratified).

The P25 and P10 strategies resulted in further decreases of the waiting time but an increase in the number of risk assessments needed compared with 5-yearly risk assessment.

In online supplemental figure S9 and S10 in the Supplementary Material, we showed the proportion of people that have crossed the risk threshold at the time of assessment and the waiting time to risk reassessment after crossing the threshold stratified by age and initial risk level. Compared with the benchmark strategy in the UK, our proposed assessment intervals reduced the waiting time of high-risk individuals (initial risk 7.5%–10%) to a third.

## Discussion

In this paper, we quantified the expected time of crossing a predefined CVD risk threshold stratified by age and initial risk level, and identified optimal risk assessment intervals for primary prevention of cardiovascular disease. To our knowledge, this is the first large-scale study seeking to provide empirical evidence on the optimal times to reassess CVD risk, using UK electronic primary care data from more than 2 million people which is representative of the UK population. Our results showed that a person’s initial estimated CVD risk has a large impact on the time to crossing a given risk treatment threshold and thus approaches to determining risk reassessment intervals should be based on initial risk. For those with an initial 10-year CVD risk of 7.5–10%, our results suggest risk reassessment should occur more frequently than the currently recommended 5-yearly risk reassessment approach to allow treatments to be commenced sooner (reducing waiting times to risk reassessment by more than two-thirds). For those with an initial 10-year risk <2.5%, risk reassessment could be done less frequently than every 5 years, with similar time spent waiting to have risk reassessed after passing the risk treatment threshold. Overall, this would require fewer risk reassessments. When stratified by age, shortened expected crossing times were observed for those aged above 65 years. Above the age of 75 years for women and 70 years for men, almost everyone was already above the risk treatment threshold based on their initial estimated risk. This suggests that risk reassessment may not be necessary for most older people in terms of guideline recommendations.

Our study supports previous findings that risk assessment intervals should be based on initial estimates of risk,^12 13^ and that 5-yearly risk assessments are unnecessarily frequent for many individuals and insufficiently frequent for individuals with high risk. The previous studies did not provide empirical data on the times to cross a given risk treatment threshold limited by the study design where data were not collected between two follow-up time points (eg, between 3 years and 8 years in Bell *et al*,^12^ and between the 5-year follow-ups in Lindbohm *et al*^13^). Our study complements these studies by providing the distribution of crossing times for each risk group.

The strengths of this study are threefold. First, the method we applied uses sparse, unbalanced repeated measurements of risk factors and allows for missing values inherent within routine clinical practice. It is computationally efficient and can be applied to large datasets. Second, the dataset we used to derive the results is large-scale and representative of the UK population with respect to age, sex and ethnicity.^14^ Therefore, the conclusions are directly applicable to the guidelines for the UK and similar populations. Finally, our proposed approach can be applied to populations with different demographics, by computing the average percentiles using the information of age structure at any given time point, and to health systems with different levels of resource abundance, by selecting a reasonable percentile.

The study also has several limitations. First, time-dependent risk factors were assumed linear over time due to the limited number of repeated measurements per individual. For studies with more data, more complex longitudinal models can be fitted. Second, we assumed that all time-dependent risk factors jointly follow a multivariate normal distribution, which was plausible for BMI, SBP, total cholesterol and HDL cholesterol but less plausible for smoking status which was defined as a binary variable. However, inference from the multivariate normal distribution may often be reasonable even if multivariate normality does not hold.^24^ Third, as we were limited by the maximum follow-up time of 15 years, we were not able to directly assess 10-year risk up to 10 years. Instead, we estimated the correspondence between the 10-year and 5-year risk and approximated 10-year risk using 5-year risk. The approximation was performed separately for each sex, landmark age and initial risk level to take into account the difference in the 10-year to 5-year risk ratios within each subgroup.

In conclusion, this study suggests that CVD risk assessment intervals should be set based on the initial risk level. For people with low risk, the interval can be prolonged safely, while for those with high risk, the interval should be much shorter. These findings are consistent with previous studies and should be incorporated into future guidelines for CVD risk assessment.

## Supplementary material

10.1136/bmjph-2024-001241 online supplemental file 1

## Data Availability

Data may be obtained from a third party and are not publicly available.
