# Supplementary material for: Optimal risk assessment intervals for primary prevention of cardiovascular disease: a population-based two-stage landmarking study
Source: BMJ Public Health. 2025 Mar 28;3(1):e001241. doi: 10.1136/bmjph-2024-001241 (PMC11956352; doi:10.1136/bmjph-2024-001241)
Supplement: online supplemental file 1 [file bmjph-3-1-s001.docx]

**Supplementary Material of: Optimal risk assessment intervals for primary prevention of cardiovascular disease: a population-based two-stage landmarking study**

Zander Gu*, Francesca Gasperoni*, Ellie Paige, Michael Sweeting, Juliet Usher-Smith, Katrina Poppe, David Stevens, Matthew Arnold, Emanuele Di Angelantonio, Angela M Wood, Jessica K Barrett

*Joint first authors

# Table of Contents

[Table of Contents 1](#_Toc184371170)

[Methods 2](#_Toc184371171)

[Cohort selection, outcome definitions and risk factors 2](#_Toc184371172)

[Extended two-stage landmarking model: stage one 12](#_Toc184371173)

[Extended two-stage landmarking model: stage two 12](#_Toc184371174)

[The 10-year to 5-year risk ratio 13](#_Toc184371175)

[The expected crossing time 13](#_Toc184371176)

[Averaging the percentiles of expected crossing times 13](#_Toc184371177)

[Risk assessment strategy evaluation metrics 14](#_Toc184371178)

[*Expected number of follow-up assessments* 14](#_Toc184371179)

[*Average waiting time* 14](#_Toc184371180)

[Results 14](#_Toc184371181)

[Estimates of Multivariate Linear Mixed Effect Model parameters 14](#_Toc184371182)

[Estimates of the Cox proportional hazard model 16](#_Toc184371183)

[Proportion of individuals crossing the risk threshold at the first risk assessment 21](#_Toc184371184)

[Average waiting time of the individuals to the first risk assessment 23](#_Toc184371185)

[References 24](#_Toc184371186)

# Methods

We applied the extended 2-stage landmarking approach to dynamically estimate the 5-year CVD risk and to identify the expected time when the 5-year CVD risk exceeds the threshold of 5%.^1–3^ The sex- and risk-specific percentiles of the expected crossing times were then averaged across the landmark ages to get a population-level summary. Several risk assessment strategies were recommended based on these percentiles and compared using various metrics.

## Cohort selection, outcome definitions and risk factors

In Figure S1, we represent the scheme of the cohort selection.

17,251,881

11,997,921

2,538,731

2,525,156

2,154,089

2,080,115

People whose data is marked as unacceptable, gender is not male or female, outside of England.
**N = 5,253,960**

Reason for exclusion: Removing people who exited before or at the start of the study or age 40, or who started after the study exit or age 85.

Study entry date was the latest of:

- The date of 6 months after the individual registered at a general practice
- The date that the individual turned 30 years of age
- The date that the data for the practice were up to standard (UTS)
- The date for enhanced data quality usage in English general practice, which was defined as the time that the national Quality and Outcomes Framework(QOF) was introduced (1st April 2004)

Study exit date was the earliest of:

- the date of deregistration at the practice
- the individual's death
- the date that the individual turned 95 years of age
- the last contact date for the practice with CPRD
- the administration end date (31st May 2019)

**N = 9,459,190**

Reason for exclusion: Removing people for whom linkage data is not available.
**N = 13,575**

Reason for exclusion: Removing people with prevalent cardiovascular disease (CVD) or statin treatment before study entry.
**N = 371,067**

People have no detected exposures of body mass index (BMI), systolic blood pressure (SBP), total cholesterol, high-density lipoprotein (HDL) cholesterol, smoking status, before study exit or a CVD event.
**N = 73,974**

Figure S1 Flow chart for cohort definition

The main outcome of interest was the first non-fatal or fatal cardiovascular event defined as a combination of newly diagnoses of nonfatal or fatal events of coronary heart disease (CHD) (including myocardial infarction and angina), stroke, and transient ischemic attack (TIA), in line with the definition used in the QRISK3 CVD risk score (code list in Table S1).^4–6^

We quantified CVD risk using risk factors included in the QRISK2/3 equations.^5,6^ Specifically, we included: body mass index (BMI) (kg/m2), systolic blood pressure (SBP) (mm Hg), total cholesterol (mmol/L), high-density lipoprotein (HDL) cholesterol (mmol/L), smoking status (current smoker or not), level of deprivation measured using the Townsend score,^7^ blood pressure medication prescription (yes/no ascertained from CPRD medication prescription information), and previous diagnoses of: diabetes, renal disease, migraine, rheumatoid arthritis, depression, severe mental illness, atrial fibrillation. The disease diagnoses are ascertained from the hierarchical read code system In Clinical Practice Research Datalink (CPRD),^6^ and the International Classification of Disease 10th revision (ICD-10) codes in the linked HES and ONS datasets.^8^ In addition, we defined statin initiation as the date of first prescription recorded in the primary care records.^9^

The following biologically implausible risk-factor values were set to missing: BMI > 80 kg/m2; SBP >250 mm Hg or <60 mm Hg; total cholesterol >20 mmol/L or <1·75 mmol/L; HDL cholesterol >3·1 mmol/L or <0·3 mmol/L. The Townsend score was missing in 0.44% of the individuals, which was imputed by the median of the cohort.

The counts of individuals by the number of measured exposure types of the time-dependent risk factors and by the number of measurements for each risk factor are shown in Figure S2 and Figure S3, respectively.

Figure S3 Count of individuals by the number of measurements for each time-dependent risk factor.


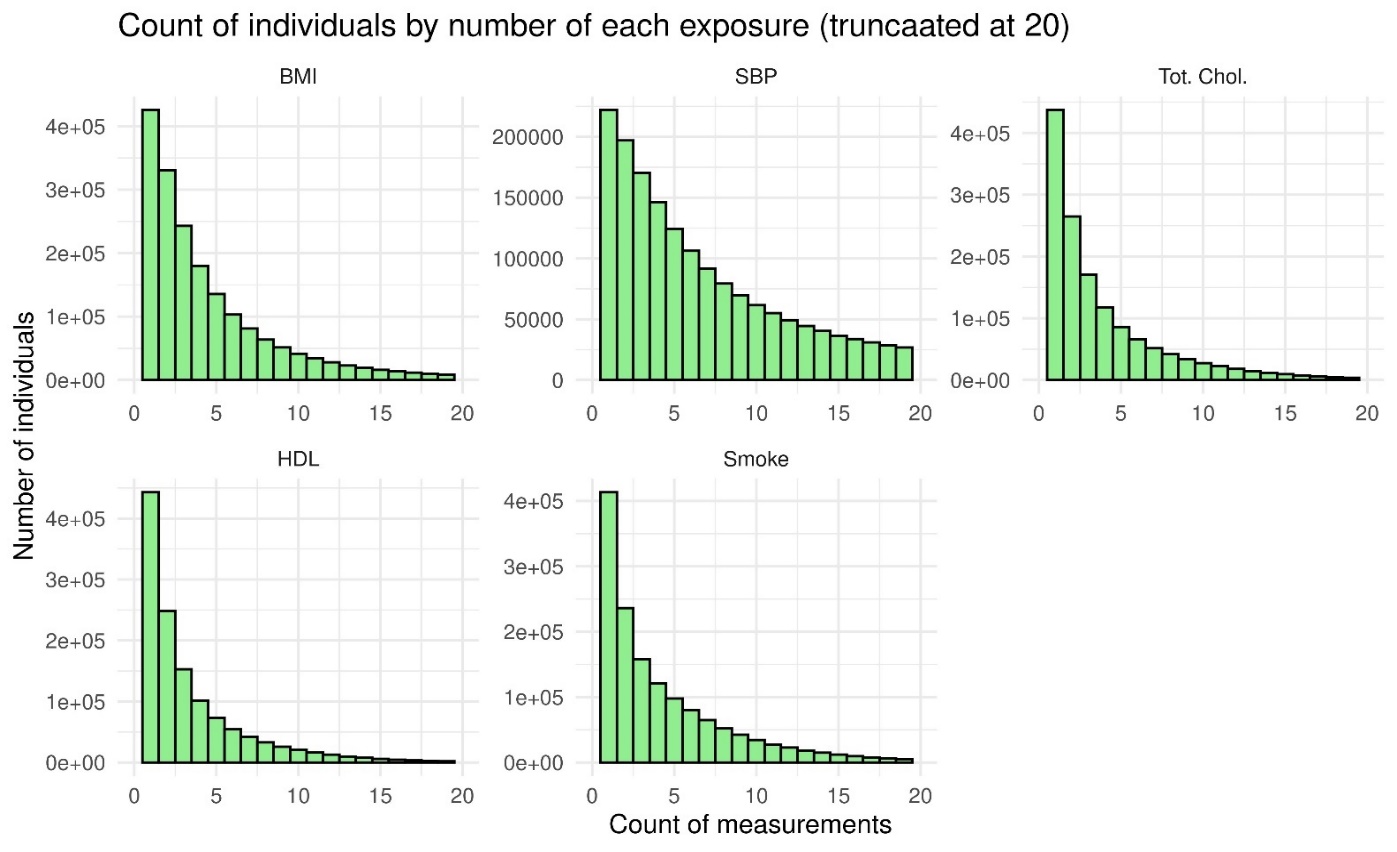

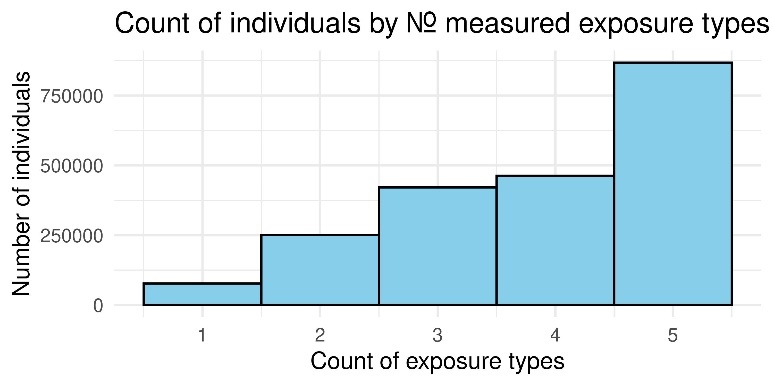


Figure S2 Count of individuals by the number of measured exposure types of the time-dependent risk factors.

Table S1 Code list of cardiovascular disease

| **Read code for CPRD data** | |
| --- | --- |
| **Read code** | **Description** |
| G3...00 | Ischaemic heart disease |
| G31..00 | Arteriosclerotic heart disease |
| G32..00 | Atherosclerotic heart disease |
| G33..00 | IHD - Ischaemic heart disease |
| G30..00 | Acute myocardial infarction |
| G301.00 | Attack - heart |
| G302.00 | Coronary thrombosis |
| G303.00 | Cardiac rupture following myocardial infarction (MI) |
| G304.00 | Heart attack |
| G305.00 | MI - acute myocardial infarction |
| G306.00 | Thrombosis - coronary |
| G307.00 | Silent myocardial infarction |
| G309800 | Coronary thrombosis |
| G309900 | Myocardial Infarction |
| G300.00 | Acute anterolateral infarction |
| G301.00 | Other specified anterior myocardial infarction |
| G301000 | Acute anteroapical infarction |
| G301100 | Acute anteroseptal infarction |
| G301z00 | Anterior myocardial infarction NOS |
| G302.00 | Acute inferolateral infarction |
| G303.00 | Acute inferoposterior infarction |
| G304.00 | Posterior myocardial infarction NOS |
| G305.00 | Lateral myocardial infarction NOS |
| G306.00 | True posterior myocardial infarction |
| G307.00 | Acute subendocardial infarction |
| G307000 | Acute non-Q wave infarction |
| G307100 | Acute non-ST segment elevation myocardial infarction |

| G308.00 | Inferior myocardial infarction NOS |
| --- | --- |
| G309.00 | Acute Q-wave infarct |
| G30A.00 | Mural thrombosis |
| G30B.00 | Acute posterolateral myocardial infarction |
| G30X.00 | Acute transmural myocardial infarction of unspecif site |
| G30X000 | Acute ST segment elevation myocardial infarction |
| G30y.00 | Other acute myocardial infarction |
| G30y000 | Acute atrial infarction |
| G30y100 | Acute papillary muscle infarction |
| G30y200 | Acute septal infarction |
| G30yz00 | Other acute myocardial infarction NOS |
| G30z.00 | Acute myocardial infarction NOS |
| G31..00 | Other acute and subacute ischaemic heart disease |
| G319900 | Acute/subacute IHD NOS |
| G310.00 | Postmyocardial infarction syndrome |
| G310100 | Dressler's syndrome |
| G311.00 | Preinfarction syndrome |
| G311100 | Crescendo angina |
| G311200 | Impending infarction |
| G311300 | Unstable angina |
| G311400 | Angina at rest |
| G311000 | Myocardial infarction aborted |
| G311010 | MI - myocardial infarction aborted |
| G311100 | Unstable angina |
| G311200 | Angina at rest |
| G311300 | Refractory angina |
| G311400 | Worsening angina |
| G311500 | Acute coronary syndrome |
| G311z00 | Preinfarction syndrome NOS |
| G312.00 | Coronary thrombosis not resulting in myocardial infarction |

| G31y.00 | Other acute and subacute ischaemic heart disease |
| --- | --- |
| G31y000 | Acute coronary insufficiency |
| G31y099 | Acute coronary syndrome |
| G31y100 | Microinfarction of heart |
| G31y200 | Subendocardial ischaemia |
| G31y300 | Transient myocardial ischaemia |
| G31yz00 | Other acute and subacute ischaemic heart disease NOS |
| G32..00 | Old myocardial infarction |
| G321.00 | Healed myocardial infarction |
| G322.00 | Personal history of myocardial infarction |
| G33..00 | Angina pectoris |
| G330.00 | Angina decubitus |
| G330000 | Nocturnal angina |
| G330z00 | Angina decubitus NOS |
| G331.00 | Prinzmetal's angina |
| G331100 | Variant angina pectoris |
| G332.00 | Coronary artery spasm |
| G33z.00 | Angina pectoris NOS |
| G33z000 | Status anginosus |
| G33z100 | Stenocardia |
| G33z200 | Syncope anginosa |
| G33z300 | Angina on effort |
| G33z400 | Ischaemic chest pain |
| G33z500 | Post infarct angina |
| G33z600 | New onset angina |
| G33z700 | Stable angina |
| G33zz00 | Angina pectoris NOS |
| G34..00 | Other chronic ischaemic heart disease |
| G349900 | Chr. ischaemic heart dis. NOS |
| G340.00 | Coronary atherosclerosis |
| G340100 | Triple vessel disease of the heart |

| G340200 | Coronary artery disease |
| --- | --- |
| G340000 | Single coronary vessel disease |
| G340100 | Double coronary vessel disease |
| G342.00 | Atherosclerotic cardiovascular disease |
| G343.00 | Ischaemic cardiomyopathy |
| G344.00 | Silent myocardial ischaemia |
| G34y.00 | Other specified chronic ischaemic heart disease |
| G34y000 | Chronic coronary insufficiency |
| G34y100 | Chronic myocardial ischaemia |
| G34yz00 | Other specified chronic ischaemic heart disease NOS |
| G34z.00 | Other chronic ischaemic heart disease NOS |
| G34z000 | Asymptomatic coronary heart disease |
| G35..00 | Subsequent myocardial infarction |
| G350.00 | Subsequent myocardial infarction of anterior wall |
| G351.00 | Subsequent myocardial infarction of inferior wall |
| G353.00 | Subsequent myocardial infarction of other sites |
| G35X.00 | Subsequent myocardial infarction of unspecified site |
| G36..00 | Certain current complication follow acute myocardial infarct |
| G360.00 | Haemopericardium/current comp follow acute myocardial infarct |
| G361.00 | Atrial septal defect/curr comp follow acute myocardial infarct |
| G362.00 | Ventricular septal defect/curr comp follow acute myocardial infarction |
| G363.00 | Ruptur cardiac wall w'out haemopericard/cur comp follow ac MI |
| G364.00 | Ruptur chordae tendinae/curr comp follow acute myocardial infarct |
| G365.00 | Rupture papillary muscle/curr comp follow acute myocardial infarct |
| G366.00 | Thrombosis atrium, auric append&vent/curr comp follow acute MI |
| G38..00 | Postoperative myocardial infarction |
| G380.00 | Postoperative transmural myocardial infarction anterior wall |
| G381.00 | Postoperative transmural myocardial infarction inferior wall |
| G382.00 | Postoperative transmural myocardial infarction other sites |
| G383.00 | Postoperative transmural myocardial infarction unspec site |
| G384.00 | Postoperative subendocardial myocardial infarction |

| G38z.00 | Postoperative myocardial infarction, unspecified |
| --- | --- |
| G3y..00 | Other specified ischaemic heart disease |
| G3z..00 | Ischaemic heart disease NOS |
| G501.00 | Post infarction pericarditis |
| Gyu3400 | [X]Acute transmural myocardial infarction of unspecif site |
| F423600 | Amaurosis fugax |
| Fyu5500 | [X]Other transnt cerebral ischaemic attacks+related syndromes |
| G63y000 | Cerebral infarct due to thrombosis of precerebral arteries |
| G63y100 | Cerebral infarction due to embolism of precerebral arteries |
| G64..00 | Cerebral arterial occlusion |
| G641.00 | CVA - cerebral artery occlusion |
| G642.00 | Infarction - cerebral |
| G643.00 | Stroke due to cerebral arterial occlusion |
| G640.00 | Cerebral thrombosis |
| G640000 | Cerebral infarction due to thrombosis of cerebral arteries |
| G641.00 | Cerebral embolism |
| G641100 | Cerebral embolus |
| G641000 | Cerebral infarction due to embolism of cerebral arteries |
| G64z.00 | Cerebral infarction NOS |
| G64z100 | Brainstem infarction NOS |
| G64z200 | Cerebellar infarction |
| G64z990 | Cerebral A. occlusion NOS |
| G64z000 | Brainstem infarction |
| G64z100 | Wallenberg syndrome |
| G64z110 | Lateral medullary syndrome |
| G64z200 | Left sided cerebral infarction |
| G64z300 | Right sided cerebral infarction |
| G64z400 | Infarction of basal ganglia |
| G65..00 | Transient cerebral ischaemia |
| G651.00 | Drop attack |
| G652.00 | Transient ischaemic attack |

| G653.00 | Vertebro-basilar insufficiency |
| --- | --- |
| G659900 | Transient Ischaemic Attacks |
| G650.00 | Basilar artery syndrome |
| G650100 | Insufficiency - basilar artery |
| G652.00 | Subclavian steal syndrome |
| G653.00 | Carotid artery syndrome hemispheric |
| G654.00 | Multiple and bilateral precerebral artery syndromes |
| G656.00 | Vertebrobasilar insufficiency |
| G65y.00 | Other transient cerebral ischaemia |
| G65z.00 | Transient cerebral ischaemia NOS |
| G65z990 | Transient Ischaemic Attacks |
| G65z000 | Impending cerebral ischaemia |
| G65z100 | Intermittent cerebral ischaemia |
| G65zz00 | Transient cerebral ischaemia NOS |
| G66..00 | Stroke and cerebrovascular accident unspecified |
| G661.00 | CVA unspecified |
| G662.00 | Stroke unspecified |
| G663.00 | CVA - Cerebrovascular accident unspecified |
| G669800 | Stroke/CVA - undefined |
| G669900 | Stroke |
| G667.00 | Left sided CVA |
| G668.00 | Right sided CVA |
| G676000 | Cereb infarct due cerebral venous thrombosis, nonpyogenic |
| G6W..00 | Cereb infarct due unspcf occlus/stenos precerebr arteries |
| G6X..00 | Cerebrl infarctn due/unspcf occlusn or sten/cerebrl artrs |
| Gyu6300 | [X]Cerebrl infarctn due/unspcf occlusn or sten/cerebrl artrs |
| Gyu6400 | [X]Other cerebral infarction |
| Gyu6500 | [X]Occlusion and stenosis of other precerebral arteries |
| Gyu6600 | [X]Occlusion and stenosis of other cerebral arteries |
| ZV12D00 | [V]Personal history of transient ischaemic attack |

| **ICD10 code for HES and ONS data** | |
| --- | --- |
| **ICD10-code** | **description** |
| G45 | transient ischaemic attack and related syndromes |
| G45.0 | transient ischaemic attack and related syndromes |
| G45.1 | transient ischaemic attack and related syndromes |
| G45.2 | transient ischaemic attack and related syndromes |
| G45.3 | transient ischaemic attack and related syndromes |
| G45.4 | transient ischaemic attack and related syndromes |
| G45.8 | transient ischaemic attack and related syndromes |
| G45.9 | transient ischaemic attack and related syndromes |
| I20 | angina pectoris |
| I20.0 | angina pectoris |
| I20.1 | angina pectoris |
| I20.8 | angina pectoris |
| I20.9 | angina pectoris |
| I21 | acute myocardial infarction |
| I21.0 | acute myocardial infarction |
| I21.1 | acute myocardial infarction |
| I21.2 | acute myocardial infarction |
| I21.3 | acute myocardial infarction |
| I21.4 | acute myocardial infarction |
| I21.9 | acute myocardial infarction |
| I22 | subsequent myocardial infarction |
| I22.0 | subsequent myocardial infarction |
| I22.1 | subsequent myocardial infarction |
| I22.8 | subsequent myocardial infarction |
| I22.9 | subsequent myocardial infarction |
| I23 | complications after myocardial infarction |
| I23.0 | complications after myocardial infarction |
| I23.1 | complications after myocardial infarction |
| I23.2 | complications after myocardial infarction |
| I23.3 | complications after myocardial infarction |
| I23.4 | complications after myocardial infarction |
| I23.5 | complications after myocardial infarction |
| I23.6 | complications after myocardial infarction |
| I23.8 | complications after myocardial infarction |
| I24 | other acute ischaemic heart disease |
| I24.0 | other acute ischaemic heart disease |
| I24.1 | other acute ischaemic heart disease |
| I24.8 | other acute ischaemic heart disease |
| I24.9 | other acute ischaemic heart disease |
| I25 | chronic ischaemic heart disease |
| I25.0 | chronic ischaemic heart disease |
| I25.1 | chronic ischaemic heart disease |
| I25.2 | chronic ischaemic heart disease |
| I25.3 | chronic ischaemic heart disease |
| I25.4 | chronic ischaemic heart disease |
| I25.5 | chronic ischaemic heart disease |
| I25.6 | chronic ischaemic heart disease |
| I25.8 | chronic ischaemic heart disease |
| I25.9 | chronic ischaemic heart disease |
| I63 | cerebral infarction |
| I63.0 | cerebral infarction |
| I63.1 | cerebral infarction |
| I63.2 | cerebral infarction |
| I63.3 | cerebral infarction |
| I63.4 | cerebral infarction |
| I63.5 | cerebral infarction |
| I63.6 | cerebral infarction |
| I63.8 | cerebral infarction |
| I63.9 | cerebral infarction |
| I64 | stroke not specified as haemorrhage or infarction |

## Extended two-stage landmarking model: stage one

A landmark cohort for each age in (40, 45,…, 80, denoted as “landmark ages $L_{a}$”) is specified as individuals who: 1) are in the study at the landmark age; 2) have not had a CVD event prior to the landmark age; 3) have at least 1 measurement of one of BMI, SBP, total cholesterol, HDL cholesterol, and smoking status; and 4) have no statin prescription prior to the landmark age. On each landmark cohort, we fitted sex-specific multivariate linear mixed effect model (LMEMs) with random intercept and random slope of age. The outcomes of the LMEMs are the time-dependent risk factors (BMI, SBP, total cholesterol, HDL cholesterol, and smoking status). Values of BMI, SBP, total cholesterol, and HDL cholesterol were standardized using sex-specific means and standard deviations. For SBP and total cholesterol, we included blood pressure medication (BPM) and statin prescription (initiated after the landmark age) as covariates, respectively. Let $BMI_{ij}, SBP_{ij}, TCHOL_{ij}, HDL_{ij}, smoke_{ij}$ be the $j$-th repeated measurement of the corresponding outcome for individual $i$, and $BPM_{ij}, statin_{ij}, age_{ij}$ denote the blood pressure-lowering medication status, statin prescription status and age at the time point of the $j$-th measurement of an outcome for individual $i$. The multivariate LMEM is given by

$BMI_{ij}= \beta_{10} + \beta_{11} age_{ij} + u_{10i} + u_{11i}age_{ij} + \varepsilon_{1ij}$,

$$SBP_{ij} = \beta_{20} + \beta_{21}age_{ij} + \beta_{22} BPM_{ij} + u_{20i} + u_{21i}age_{ij} + \varepsilon_{2ij},$$

$TCHOL_{ij} = \beta_{30} + \beta_{31} age_{ij} + \beta_{32} statin_{ij} + u_{30i} + u_{31i}age_{ij} + \varepsilon_{3ij}$,

$HDL_{ij} = \beta_{40} + \beta_{41} age_{ij} + u_{40i} +u_{41i}age_{ij} + \varepsilon_{4ij}$,

$smoke_{ij} = \beta_{50} + \beta_{51} age_{ij} + u_{50i} + u_{51i}age_{ij} + \varepsilon_{5ij}$.

Here, $\boldsymbol{\beta}_{\mathbf{0}}=(\beta_{10}, \beta_{20},\beta_{30},\beta_{40},\beta_{50})$ represents fixed intercepts and $\boldsymbol{\beta}_{\mathbf{1}}=(\beta_{11}, \beta_{21},\beta_{31},\beta_{41},\beta_{51})$ represents fixed slope, for each risk factor. $\beta_{22}$ represents an adjustment factor in systolic blood pressure levels for those subjects under blood-pressure lowering medication at the time the measurement was taken. $\beta_{32}$ is the regression parameter that represents the effect of statin prescription on total cholesterol. The random intercepts and random slopes $\boldsymbol{u}_{0}=(u_{10}, u_{20},u_{30},u_{40},u_{50})$ and $\boldsymbol{u}_{1}=(u_{11}, u_{21},u_{31},u_{41},u_{51})$ follow a multivariate normal distribution: $\binom{\boldsymbol{u}_{0}}{\boldsymbol{u}_{1}}\sim N(\mathbf{0}, \Sigma)$. The residuals $\boldsymbol{\varepsilon}_{\boldsymbol{ij}}=(\varepsilon_{1ij}, \varepsilon_{2ij},\varepsilon_{3ij},\varepsilon_{4ij},\varepsilon_{5ij})$ are independent of $\boldsymbol{u}_{\mathbf{0}}, \boldsymbol{u}_{\mathbf{1}}$, and of each other.

For each landmark age $L_{a}$, we predicted the time-dependent risk factors at $L_{a}$ and at each year after $L_{a}$ up to 10 years (i.e., $L_{a}$, $L_{a}+1, L_{a}+2, \ldots, L_{a}+10$, denoted as “times of interest”) as the best linear unbiased predictors (BLUPs) from the corresponding fitted LMEM. Note that the prediction essentially imputes missing values at the time of risk prediction using past measurements of all time-dependent risk factors. This prediction of time-dependent risk factors relies on the variance-covariance structure of the random effects, thus it is not required to have measurements of all risk factors for each individual.

## Extended two-stage landmarking model: stage two

Due to restrictions of the maximum follow-up, analysis was conducted to determine 5-year CVD risks which were then transformed to 10-year risks using age and sex-stratified models. We estimated the 5-year CVD risk at each time of interest $t$ using landmark age- and sex-specific Cox proportional hazards models, adjusting for time-dependent and time-fixed risk factors. The time-dependent risk factors are BMI, SBP, total cholesterol, HDL cholesterol, and smoking status, which entered the model via the BLUPs predicted at each time of interest at the previous stage. The time-fixed risk factors are the quintiles of the Townsend score,^7^ blood pressure medication prescription, and previous diagnoses of: diabetes, renal disease, migraine, rheumatoid arthritis, depression, severe mental illness, and atrial fibrillation, measured at the landmark age $L_{a}$. Among the time-fixed risk factors, renal disease, rheumatoid arthritis and atrial fibrillation were included only from landmark age 60 onwards, because these diagnoses were very rare at younger landmark ages. Let $\boldsymbol{x}_{i,BLUP}^{T}\left( t \right)$ be the vector of time-dependent risk factors predicted at time $t$ for individual $i$, and $\boldsymbol{x}_{i,baseline}^{T}$ be the time-fixed risk factors measured at the landmark age $L_{a}$. The Cox proportional hazard model for each time of interest $t$ is given by

$\lambda\left( s;\boldsymbol{x}_{i}\left( t \right) \right)= \lambda_{0}\left( s \right)\cdot\exp\left\{ \boldsymbol{x}_{i,baseline}^{T} \boldsymbol{\beta}_{baseline} + \boldsymbol{x}_{i,BLUP}^{T}\left( t \right) \boldsymbol{\beta}_{BLUP} \right\}$,

$$i\in\left\{ 1,..,N_{L_{a},t} \right\}, t\in\left\{ L_{a}, L_{a}+1, L_{a}+2, .., L_{a}+10 \right\},$$

$$L_{a}\in\left\{ 40, 45,..., 80 \right\}, t\leq s\leq t + 5.$$

where $\lambda_{0}\left( s \right)$ is the nonparametric baseline hazard function, $\boldsymbol{\beta}_{baseline}$ is the regression parameter vector associated to variables measured at baseline ${(L}_{a})$ and $\boldsymbol{\beta}_{BLUP}$ is the regression parameter vector associated to the BLUPs of BMI, SBP, total cholesterol, HDL cholesterol, and smoking status computed at time $t\in\{L_{a}$, $L_{a}+1$, $L_{a}+2$, .., $L_{a}+10\}$. $N_{L_{a},t}$ is the size of the landmark sub-cohort composed of those people contained in the landmark cohort of $L_{a}$ that are still alive and have not being diagnosed with CVD at time $t$ (i.e., $N_{L_{a},L_{a}}>N_{L_{a},L_{a}+1}>\ldots>N_{L_{a},L_{a}+10}$).

The 5-year CVD risk $r$for each person at each time of interest was then estimated based on the corresponding fitted Cox model as

$$\hat{r}_{i}\left( t+5;\mathbf{x}_{i}\left( t \right) \right)=1- exp\left\{ -\hat{\Lambda}_{0}\left( t+5 \right)\cdot exp\left\{ \mathbf{x}_{i}^{T}\left( t \right) \hat{\boldsymbol{\beta}} \right\} \right\} ,$$

where $\hat{\Lambda}_{0}\left( s \right)$ is the estimated cumulative baseline hazard function, $\hat{\boldsymbol{\beta}}=({\hat{\boldsymbol{\beta}}}_{baseline}$, ${\hat{\boldsymbol{\beta}}}_{BLUP})$ is the estimated vector of coefficients, and $\boldsymbol{x}_{i}^{T}(t)=( \boldsymbol{x}_{i,baseline}^{T}, \boldsymbol{x}_{i,BLUP}^{T}\left( t \right))$ is the corresponding risk factors.

## The 10-year to 5-year risk ratio

For each landmark cohort, we fitted two Cox models. One for the time from the landmark age to the first CVD event censored at 5 years, and the other censored at 10 years. The 5-year and 10-year risk for each individual in the landmark cohort was then computed from the two models respectively. The individuals were then further grouped by sex and their initial risk level (based on their predicted 10-year risk level: >10%, 7·5%-10%, 5%-7·5%, 2·5%-5%, or <2·5%), and the average ratio between the 10-year and 5-year risk was computed for each subgroup.

The predicted 5-year risks at each time of interest were then converted to 10-year risks by multiplying the corresponding 10-year to 5-year risk ratio.

## The expected crossing time

The expected crossing time $t^{*}$ was computed by linear interpolation between the first time of interest when the estimated 10-year CVD risk is higher than the risk threshold of 10% and the previous time of interest. We defined the expected crossing time to be >10 years if the 10-year CVD risk stayed below the threshold for the entire 10 years. Note that the individuals who had a predicted 10-year risk above 10% (from the 10-year Cox model) was excluded from the calculation of expected crossing times. For those included in the analysis, a small number of individuals might have an estimated 10-year risk (converted from the predicted 5-year risk) above 10% at the landmark age, thus had a crossing time of 0.

## Averaging the percentiles of expected crossing times

We first calculated the percentiles of the expected crossing times per landmark age per risk group and then averaged the percentiles across landmark ages as weighted sums. The weights were constructed using the demographics of the UK in 2020 and the relative sizes of risk groups for each landmark age from the CPRD GOLD dataset.^10^ Given a risk group $k$ (>10%, 7·5%-10%, 5%-7·5%, 2·5%-5%, or <2·5%), the weights for the percentiles of the landmark ages are proportional to the cohort sizes and sum up to 1. Let $\boldsymbol{Q}^{k}=(Q_{40}^{k}, Q_{45}^{k},\ldots, Q_{80}^{k})$ be the vector of a specific percentile (50th, 25th, 10th, or 5th) of the expected crossing times within risk group $k$ for each landmark age, $\boldsymbol{N}_{d}=(n_{40},n_{45},\ldots,n_{80})$ be the vector of population sizes (for women or men) aged 40, 45, …, 80 at a specific year $d$, and $\boldsymbol{\alpha}^{k}=(\alpha_{40}^{k},\alpha_{45}^{k},\ldots, \alpha_{80}^{k})$ be the vector of population sizes of individuals (with corresponding sex with $\boldsymbol{N}_{d}$) with risk level $k$ in each landmark cohort in the CPRD GOLD dataset. The weight vector $\boldsymbol{w}^{k}$for averaging the percentiles cross landmark ages is given by the unitized Hadamard product of $\boldsymbol{N}_{d}$and $\boldsymbol{\alpha}^{k}$,

$$\boldsymbol{w}^{k}= \frac{\boldsymbol{N}_{d}\odot\boldsymbol{\alpha}^{k}}{\|\boldsymbol{N}_{d}\odot\boldsymbol{\alpha}^{k}\|},$$

and the average percentile $\bar{Q}^{k}$is the dot product between $\boldsymbol{Q}^{k}$ and $\boldsymbol{w}^{k}$, i.e.,

$$\bar{Q}^{k}=\boldsymbol{Q}^{k}\cdot\boldsymbol{w}^{k}.$$

In this paper, $\boldsymbol{N}_{d}$ was taken as the UK population at mid-2020 from the office for National Statistics (<https://www.ons.gov.uk/peoplepopulationandcommunity/populationandmigration/populationestimates/bulletins/annualmidyearpopulationestimates/mid2020#the-uk-population-at-mid-2020>).

## Risk assessment strategy evaluation metrics

### *Expected number of follow-up assessments*

Let $\boldsymbol{w}=(w_{1}, w_{2},\ldots,w_{5})$ be vector of proportions of individuals belonging to the 5 risk groups (>10%, 7·5%-10%, 5%-7·5%, 2·5%-5%, or <2·5%) in the population, which can be computed as $\boldsymbol{w}= \frac{{(\boldsymbol{N}}_{d}\cdot\boldsymbol{\alpha}^{1}, \boldsymbol{N}_{d}\cdot\boldsymbol{\alpha}^{2}, \ldots, \boldsymbol{N}_{d}\cdot\boldsymbol{\alpha}^{5})}{\|{(\boldsymbol{N}}_{d}\cdot\boldsymbol{\alpha}^{1}, \boldsymbol{N}_{d}\cdot\boldsymbol{\alpha}^{2}, \ldots, \boldsymbol{N}_{d}\cdot\boldsymbol{\alpha}^{5})\|}.$Under the assumption that the risk structure in the population (i.e., the vector $\boldsymbol{w}$) is constant throughout the window of 10 years, the expected number of follow-up assessments per person within 10 years was computed as $\left( \frac{10}{S_{1}},\frac{10}{S_{2}},\ldots, \frac{10}{S_{5}} \right)\cdot\boldsymbol{w},$ where $S_{k}$ is the risk assessment interval for risk group $k$.

Proportion crossing the threshold before first risk assessment

Let $\boldsymbol{P}^{k}=(P_{40}^{k},P_{45}^{k},\ldots, P_{80}^{k})$ be the vector of proportions of individuals in risk group $k$ who have crossed the risk threshold before the first risk assessment $S_{k}$ in each landmark cohort (i.e., $t^{*}<S_{k}$). The average effectiveness for risk group $k$ is given by $\bar{P}^{k}=\boldsymbol{P}^{k}\cdot\boldsymbol{w}^{k}$.

### *Average waiting time*

Let $\boldsymbol{T}^{k}=(T_{40}^{k},T_{45}^{k},\ldots, T_{80}^{k})$ be the vector of the average waiting time of individuals in risk group $k$ after crossing the threshold. The average waiting time was calculated as $\bar{T}^{k}=\boldsymbol{T}^{k}\cdot\boldsymbol{w}_{cross}^{k}$, where the weight vector $\boldsymbol{w}_{cross}^{k}=\frac{\boldsymbol{N}_{d}\odot\boldsymbol{\alpha}^{k}\odot\boldsymbol{P}^{k}}{\|\boldsymbol{N}_{d}\odot\boldsymbol{\alpha}^{k}\odot\boldsymbol{P}^{k}\|}$.

# Results

## Estimates of Multivariate Linear Mixed Effect Model parameters

The estimates of the parameters ($\boldsymbol{\beta}_{0}, \boldsymbol{\beta}_{1}, \beta_{22}, \beta_{32})$ and the associated confidence intervals are reported in Figure S4. Overall, the trend of intercepts (left column) was consistent with the estimate of the age effect (central column), i.e., increasing intercept with age corresponded to a positive age effect, and vice versa. The estimates of age effect was different across different outcomes (reported in rows). For BMI, we observed an inverse trend (i.e., at lower landmark ages, the higher the age the higher was the BMI, at higher landmark ages, the higher the age the lower was the BMI). For HDL and smoking status, we recorded a vanishing trend of the age effect (the estimated age effect tended to zero as landmark age increased). The estimate of age effect on SBP and total cholesterol was similar to that on BMI, but the interpretation could be different due to the effect of blood pressure medication and statin (right column), which are expected to be used by a larger proportion of members in older groups. From the right column in the Figure S4, blood pressure medication decreased the SBP (all CI were below 0, expect for women at landmark age of 40) and analogously, statins prescription reduced the total cholesterol.


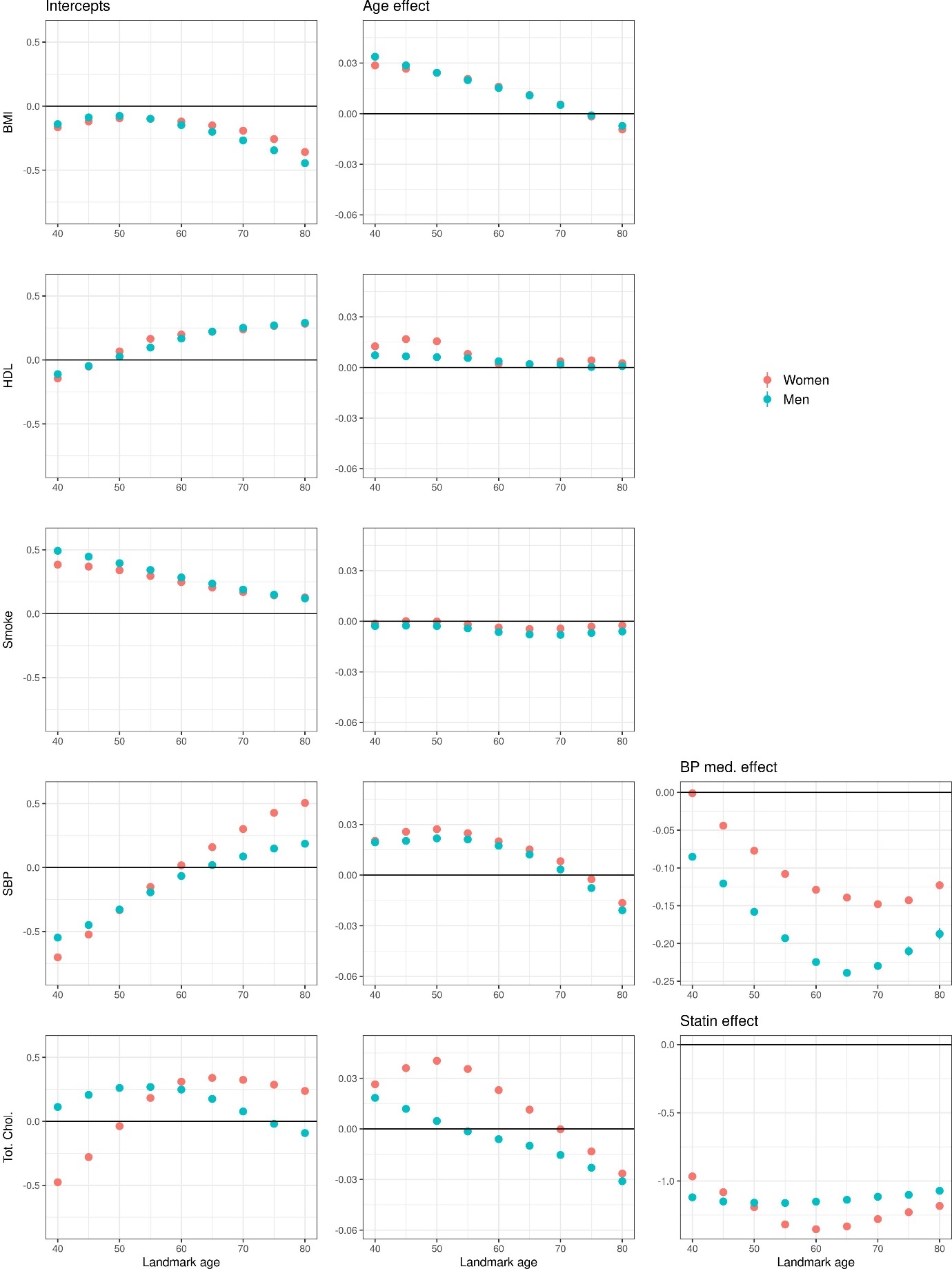


Figure S4 Estimated fixed effects of the multivariate linear mixed effect models. Each outcome of interest is reported in a row (BMI, HDL, smoke, SBP, total cholesterol from top to bottom), while intercept, slopes and other effects are reported in columns. Note that the scale of the y-axis varies across plots.

## Estimates of the Cox proportional hazard model

The hazard ratios (HRs) and the associated confidence interval for each $L_{a}\in\left\{ 40,45,..,80 \right\}$ and $t\in\left\{ L_{a}, L_{a}+1, L_{a}+2, .., L_{a}+10 \right\}$ are reported in Figure S5-Figure S7.

The HRs related to BLUPs of BMI, SBP, total cholesterol, HDL, and smoking status are shown in Figure S5. The hazard ratios for BMI (Figure S5A) were close to the null at all ages. SBP (Figure S5B), total cholesterol (Figure S5C), and smoking status (Figure S5E) were risk factors, especially below age of 70. Higher level of HDL cholesterol (Figure S5D) was associated with decreased hazard, especially for young men.

The HRs related to the time-fixed risk factors are reported in Figure S6 (factors that were considered in all models) and Figure S7 (factors that were included only in the models with landmark age over or equal to 60). Having a high Townsend score of 4 or 5 (Figure S6C-D) was a risk factor across all landmarks and a higher effect was observed in the women cohort, while having a lower Townsend score of 2 or 3 (Figure S6A-B) was not associated with higher hazard. Being prescribed blood pressure medication was associated with higher hazard (Figure S6E), possibly due to indication of higher blood pressure. Having a diagnosis of diabetes (Figure S6F) was associated with a higher hazard of being diagnosed CVD. However, we note that the confidence intervals were wide especially at younger ages. This can be explained by the very low number of diagnoses in these ages. Depression (Figure S6G) and migraine (Figure S6H) were risk factors with a higher impact on women. Also note that the prevalence of both depression and migraine was much higher in women than in men (Table 1 in the main article). Severe mental illness (Figure S6I) was not a risk factor for most of the landmark ages.

Both atrial fibrillation (Figure S7A) and rheumatoid arthritis (Figure S7B) were associated with increased hazard of CVD diagnosis. Being diagnosed with a renal disease (Figure S7C) did not represents a significantly higher risk of CVD diagnosis for most ages.

Figure S5 Hazard ratios (HR) related to BMI, SBP, total cholesterol, HDL, and smoking status are reported in panel A, B, C, D, E respectively. Each panel is composed of two plots, the one at the top shows the HR related to women, the one at the bottom to men. We represent the HR and their confidence intervals for each $t\in\left\{ L_{a}, L_{a}+1, L_{a}+2, .., L_{a}+10 \right\}$ and we associate a specific color to a specific landmark age.


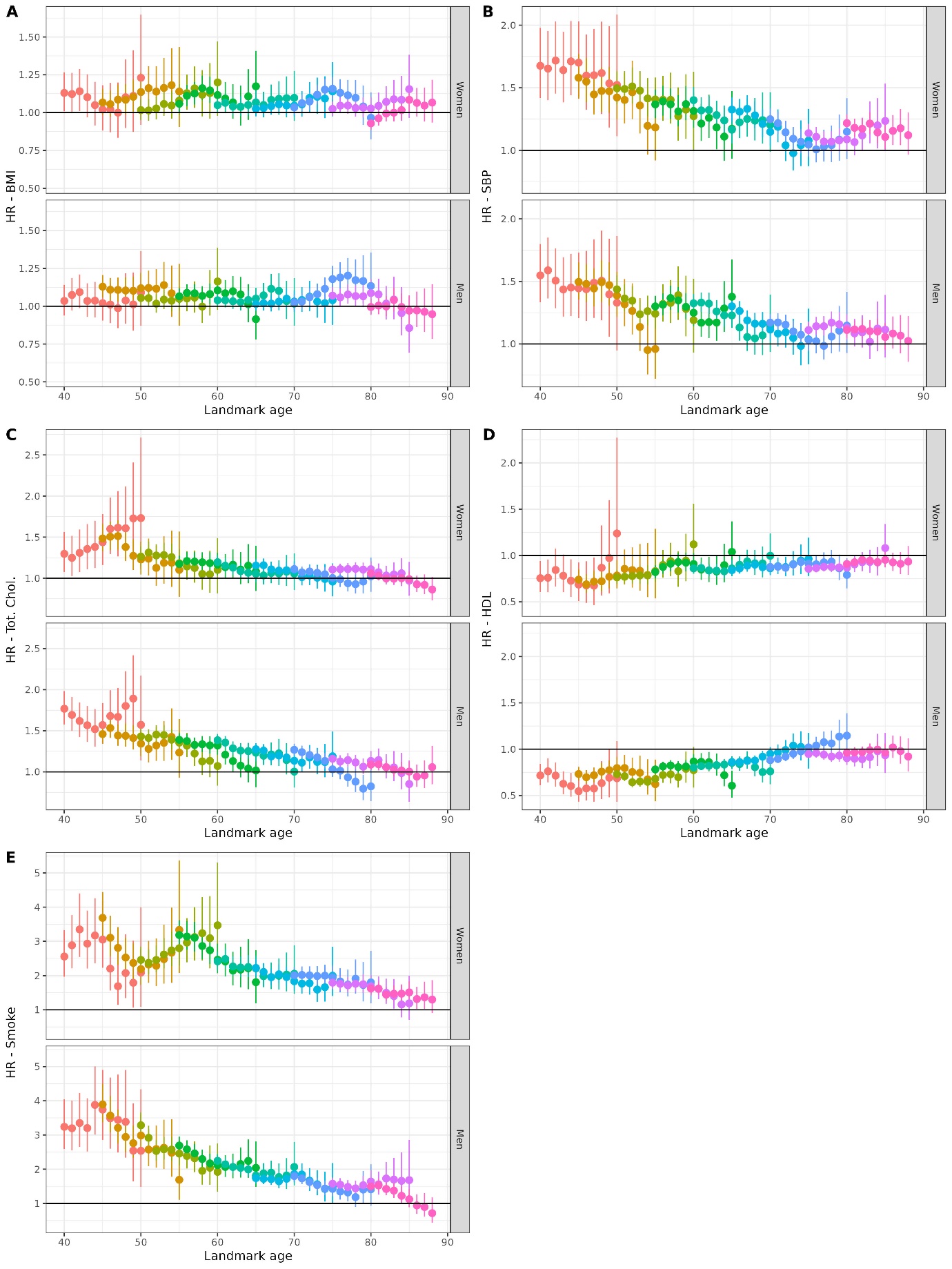


Figure S6 Hazard ratios (HR) related to Townsend deprivation score (level 2-5), blood pressure medication, diabetes, depression, migraine, severe mental illness (variables that were considered in all models) are reported in panel A-D, E, F, G, H, I, respectively. Each panel is composed of two plots, the one at the top shows the HR related to women, the one at the bottom to men. We represent the HR and their confidence intervals for each $t\in\left\{ L_{a}, L_{a}+1, L_{a}+2, .., L_{a}+10 \right\}$ and we associate a specific color to a specific landmark age.


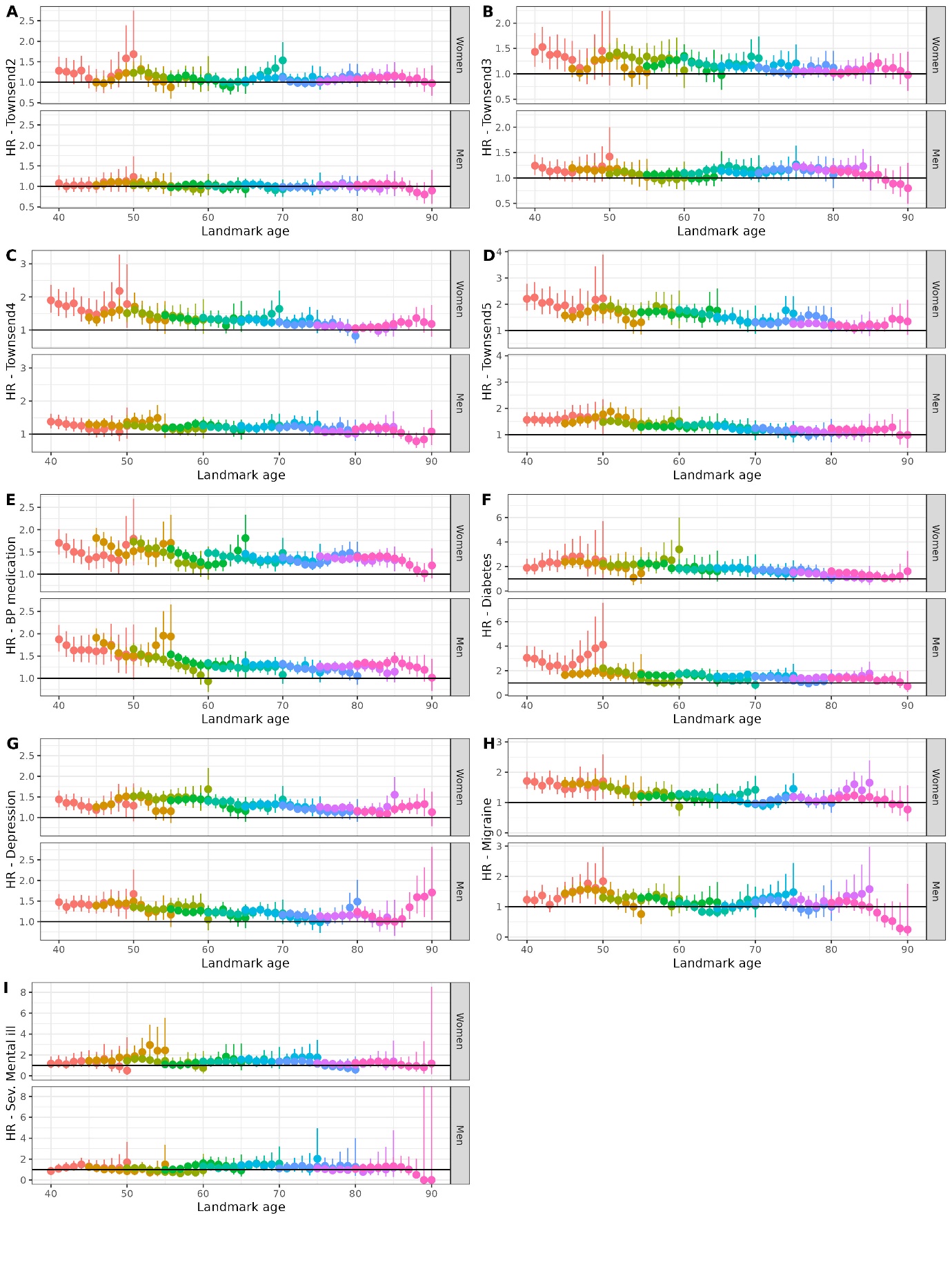


Figure S7 Hazard ratios (HR) related to atrial fibrillation, rheumatoid arthritis, chronic kidney disease are reported in panel A, B, C respectively. These variables were included only in the models with landmark age over or equal to 60. Each panel is composed of two plots, the one at the top shows the HR related to women, the one at the bottom to men. We represent the HR and their confidence intervals for each $t\in\left\{ L_{a}, L_{a}+1, L_{a}+2, .., L_{a}+10 \right\}$ and we associate a specific color to a specific landmark age.


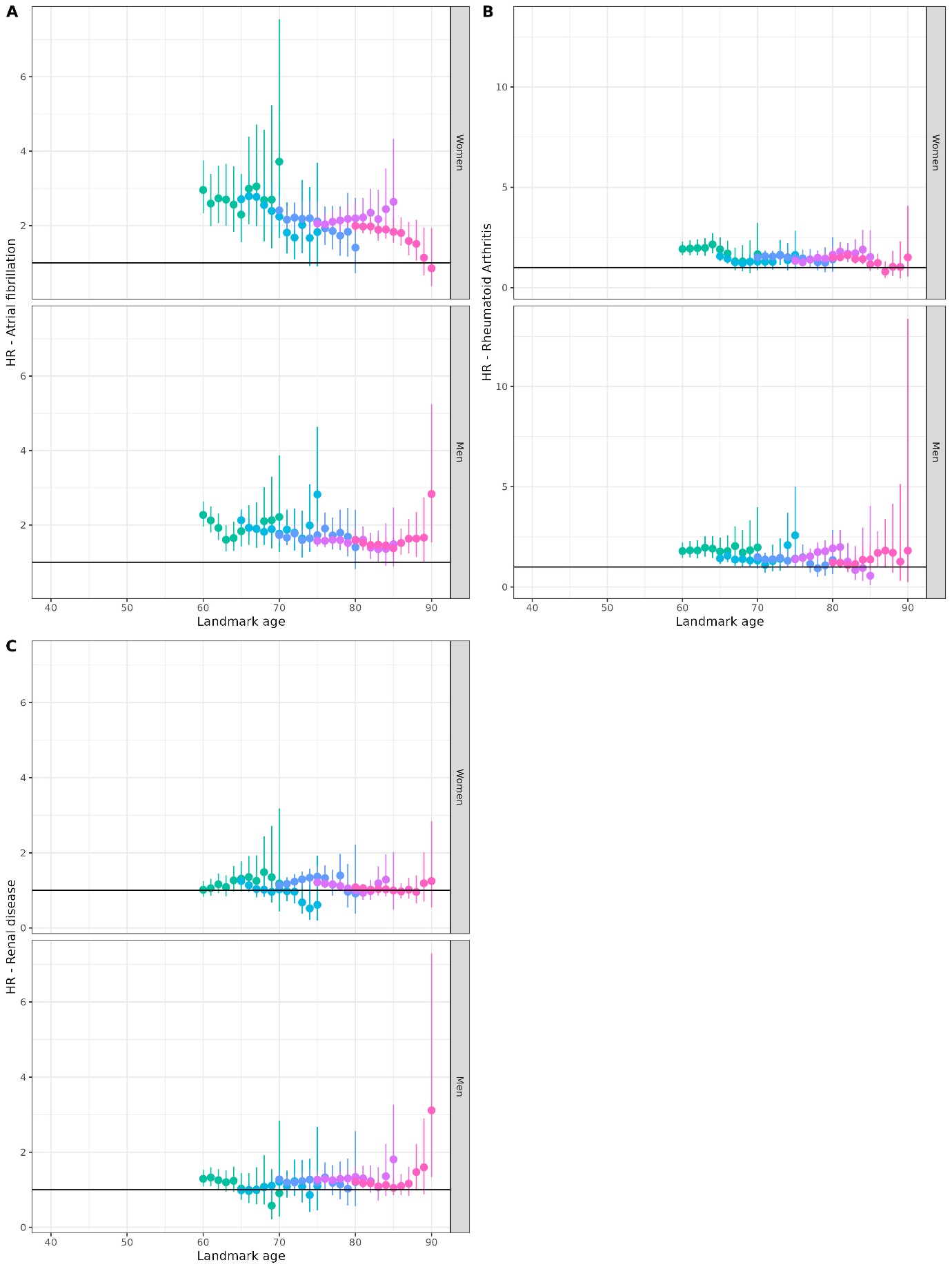


Distributions of the expected crossing times

Table S2 Number (proportion) of individuals with an initial risk greater than the risk treatment threshold of 10% 10-year risk at each landmark age

|  | Landmark age (years) | 40 | 45 | 50 | 55 | 60 | 65 | 70 | 75 | 80 |  |
| --- | --- | --- | --- | --- | --- | --- | --- | --- | --- | --- | --- |
| Women | Total | 240,534 | 242,193 | 218,230 | 186,240 | 165,081 | 129,286 | 94,296 | 69,843 | 55,415 |  |
|  | Already crossed threshold | 1,453 (0·6%) | 4,867 (2·0%) | 8,917 (4·1%) | 14,276 (7·7%) | 27,404 (16·6%) | 53,392 (41·3%) | 86,914 (92·2%) | 69,839 (100%) | 55,415 (100%) |  |
|  |  |  |  |  |  |  |  |  |  |  |  |
| Men | Total | 232,402 | 232,973 | 205,087 | 168,256 | 142,982 | 103,226 | 68,512 | 46,215 | 34,350 |  |
|  | Already crossed threshold | 3,435 (1·5%) | 15,170 (6·5%) | 42,774 (20·9%) | 71,737 S(42·6%) | 103,271 (72·2%) | 102,136 (98·9%) | 68,512 (100%) | 46,215 (100%) | 34,350 (100%) |  |

Figure S8 Average 50th(P50), 25th(P25), and 10th(P10) percentiles of expected crossing times per risk group across landmark ages for women and men. An arrow on the bar indicates that the percentile is larger than 10 years.


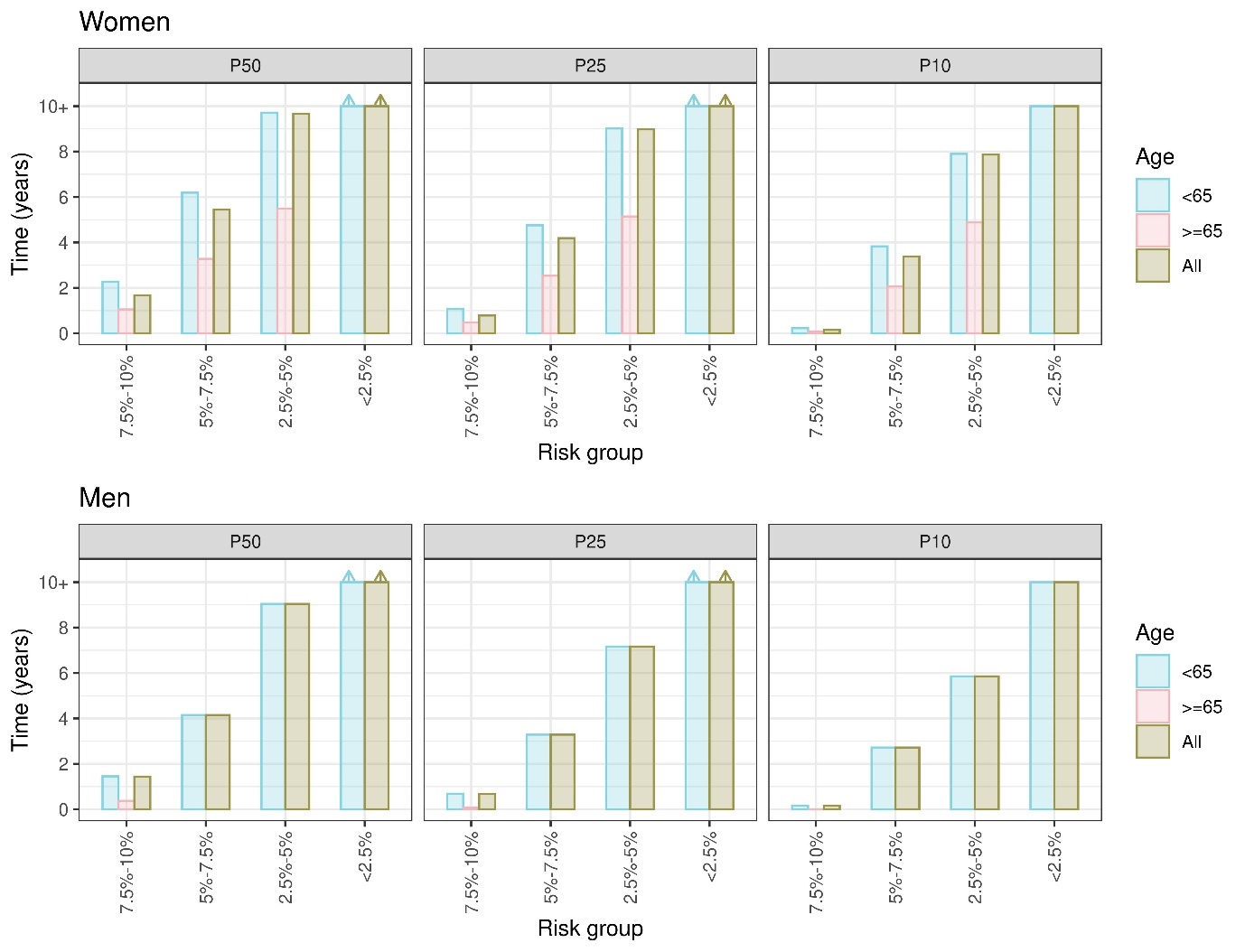


Table S3 Percentiles of the expected crossing times per sex, landmark age and risk group.

| **landmark age** | **risk group** | **Women** | | | **Men** | | |
| --- | --- | --- | --- | --- | --- | --- | --- |
|  |  | **P50** | **P25** | **P10** | **P50** | **P25** | **P10** |
| 40 | 7·5%-10% | 1.47 | 0.76 | 0 | 1.4 | 0.63 | 0 |
|  | 5%-7·5% | 4.68 | 3.79 | 3.11 | 3.82 | 3.08 | 2.47 |
|  | 2·5%-5% | 10 | 9.39 | 7.6 | 8.82 | 7.27 | 5.5 |
|  | <2·5% | 10 | 10 | 10 | 10 | 10 | 10 |
| 45 | 7·5%-10% | 1.5 | 0.64 | 0 | 1.45 | 0.75 | 0.12 |
|  | 5%-7·5% | 5.57 | 3.87 | 3.06 | 4.46 | 3.58 | 2.94 |
|  | 2·5%-5% | 9.54 | 8.59 | 7.71 | 10 | 7.73 | 6.4 |
|  | <2·5% | 10 | 10 | 10 | 10 | 10 | 10 |
| 50 | 7·5%-10% | 2.41 | 1.25 | 0.22 | 1.3 | 0.63 | 0.15 |
|  | 5%-7·5% | 6.61 | 5.03 | 4.23 | 4.06 | 3.05 | 2.43 |
|  | 2·5%-5% | 10 | 10 | 8.06 | 7.7 | 6.03 | 5.31 |
|  | <2·5% | 10 | 10 | 10 | 10 | 10 | 9.88 |
| 55 | 7·5%-10% | 1.76 | 0.8 | 0.15 | 1.7 | 0.81 | 0.24 |
|  | 5%-7·5% | 6.88 | 4.64 | 3.42 | 4.18 | 3.48 | 3 |
|  | 2·5%-5% | 10 | 9.6 | 8.69 | 6.9 | 6.1 | 5.71 |
|  | <2·5% | 10 | 10 | 10 |  |  |  |
| 60 | 7·5%-10% | 2.65 | 1.25 | 0.31 | 1.18 | 0.51 | 0.07 |
|  | 5%-7·5% | 5.78 | 4.94 | 4.13 | 3.82 | 3.28 | 2.96 |
|  | 2·5%-5% | 8.95 | 7.23 | 6.7 | 6.74 | 6.29 | 5.88 |
|  | <2·5% | 10 | 10 | 9.93 |  |  |  |
| 65 | 7·5%-10% | 1.19 | 0.56 | 0.10 | 0.37 | 0.08 | 0.00 |
|  | 5%-7·5% | 3.28 | 2.54 | 2.07 |  |  |  |
|  | 2·5%-5% | 5.49 | 5.14 | 4.89 |  |  |  |
| 70 | 7·5%-10% | 0.53 | 0.16 | 0.00 |  |  |  |
|  | 5%-7·5% | 2.38 | 2.18 | 2.02 |  |  |  |

## Proportion of individuals crossing the risk threshold at the first risk assessment

The proportion crossing the threshold before first risk assessment per risk group per landmark age group (<65, ≥ 65, and all ages) are shown in Figure S9. For the benchmark approach of re-assessing risk every 5 years, the proportions were unbalanced across risk groups, with the proportion close to 1 in 7·5%-10% group and close to 0 in 2·5%-5% and <2·5% group. By shortening the interval for the 7·5%-10% group and lengthening the interval for the 2·5%-5% and <2·5% group, the strategies based on P50 made the proportions across risk groups more balanced. Although most of the recommended strategies adopted the maximum assessment interval of 10 years for the <2·5% groups, the proportions (combining all ages) for this group were still close to 0. This suggests that the assessment interval for this risk group might be further extended beyond 10 years safely. The proportions between the <65 and ≥ 65 age groups were more balanced in age-stratified strategies compared to non-stratified strategies.

Figure S9 Proportion of individuals who had crossed the risk threshold at the first follow-up risk assessment. It was evaluated on each risk group with landmark age <65, ≥ 65, and all ages, respectively. In the columns are results of strategies based on different average percentiles. Rows are results for non-stratified and age-stratified strategies. The corresponding assessment intervals are annotated in each subplot.


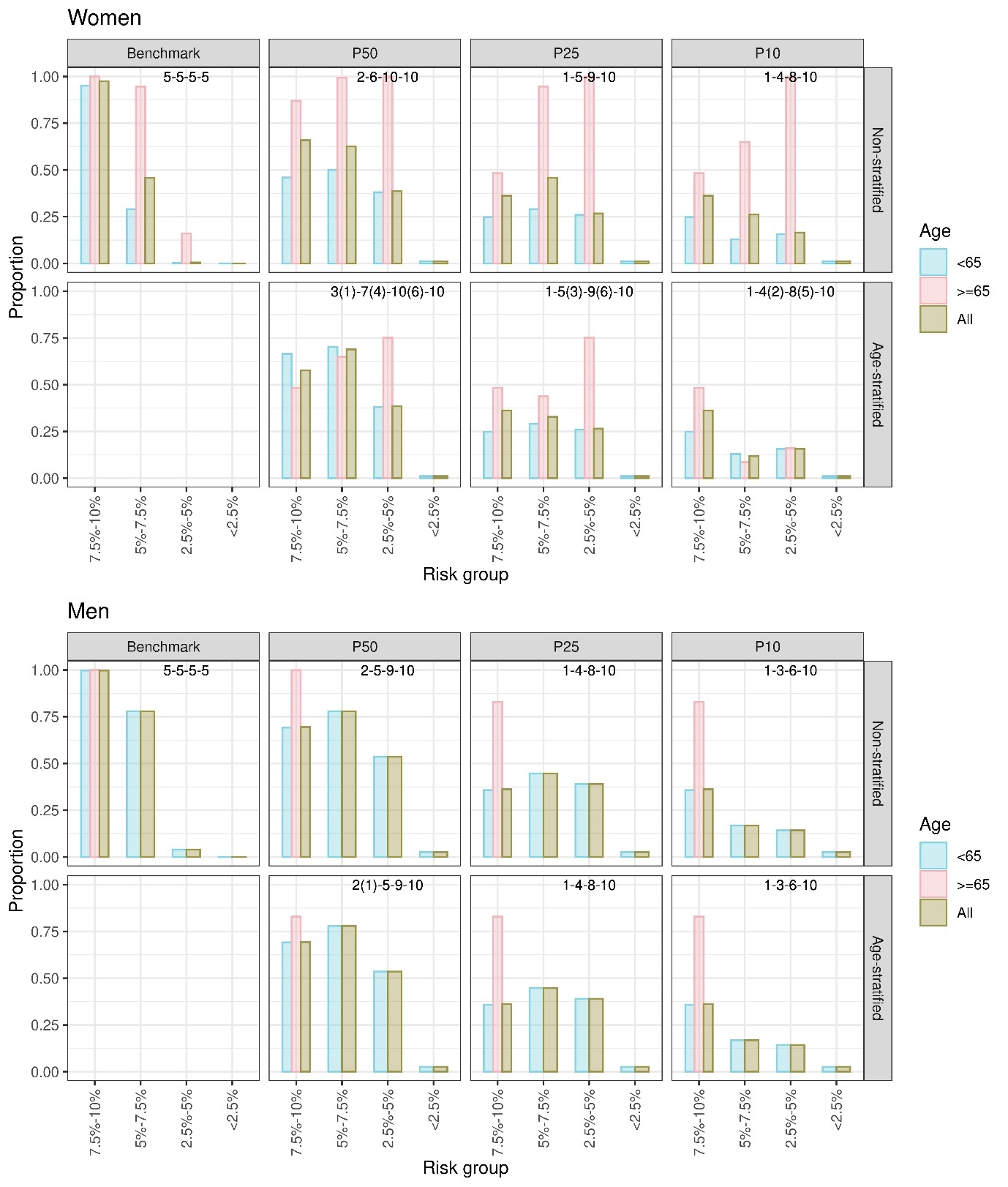


## Average waiting time of the individuals to the first risk assessment

The average waiting time per risk group per landmark age group (<65, ≥ 65, and all ages) are shown in Figure S10. The long average waiting time of over 3 years for individuals in the 7·5%-10% risk group under the benchmark approach was reduced to around 1 year under P50-based strategies and to around half a year under strategies based on smaller percentiles. In our recommended strategies which were not age-stratified, the average waiting time for women above 65 in the 5%-7·5% and 2·5%-5% risk groups was greater than 2 years in many cases. The age-stratified strategy decreased the waiting time of these older individuals to below 1 year in most of the cases.

Figure S10 Average waiting time to the first follow-up risk assessment after crossing the risk threshold. It was evaluated on each risk group with landmark age <65, ≥ 65, and all ages, respectively. In the columns are results of strategies based on different average percentiles. Rows are results for non-stratified and age-stratified strategies. The corresponding assessment intervals are annotated in each subplot.


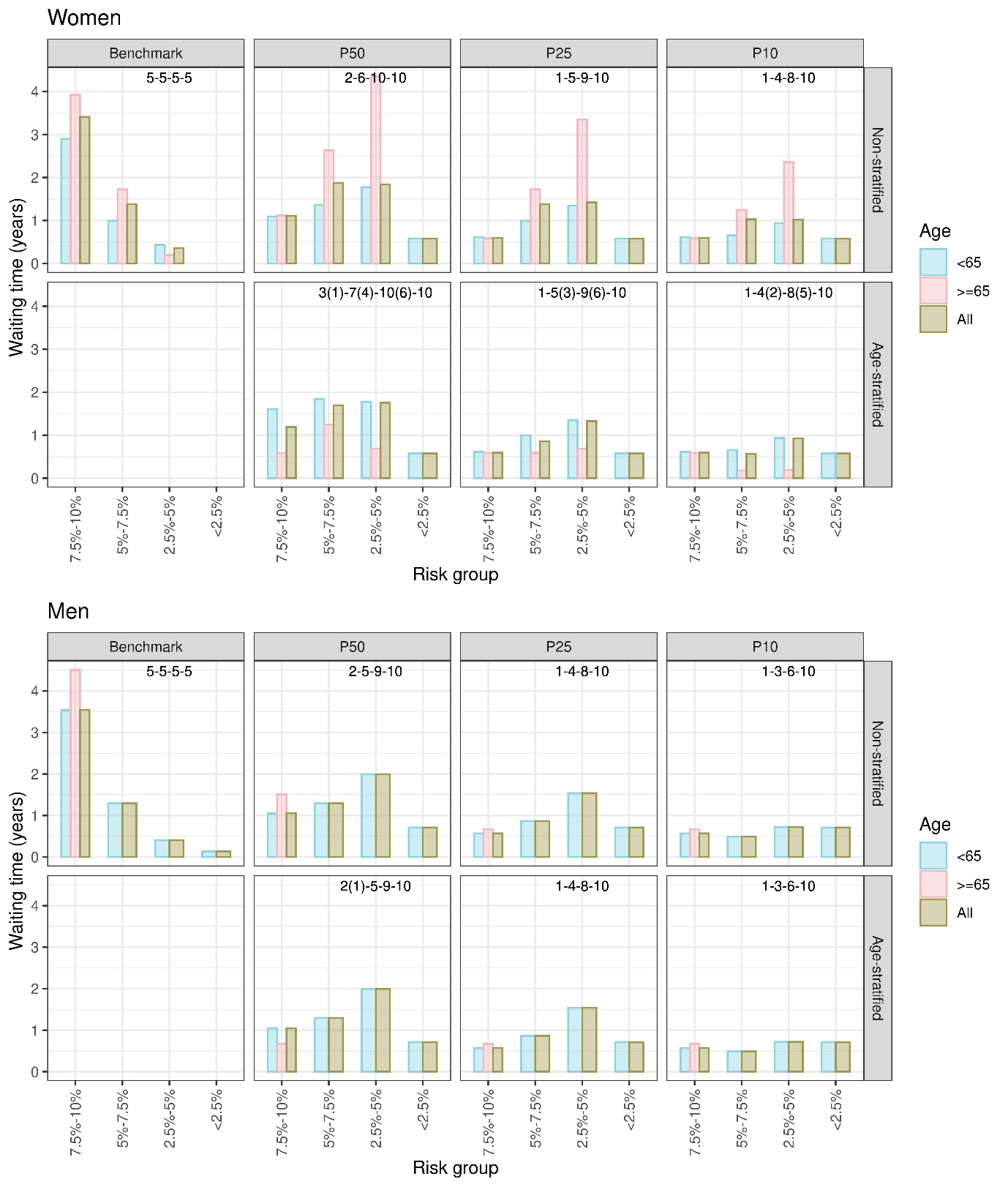


# References

1 Chung R, Xu Z, Arnold M, *et al.* Prioritising cardiovascular disease risk assessment to high risk individuals based on primary care records. *PLOS ONE* 2023; **18**: e0292240.

2 Paige E, Barrett J, Stevens D, *et al.* Landmark Models for Optimizing the Use of Repeated Measurements of Risk Factors in Electronic Health Records to Predict Future Disease Risk. *American Journal of Epidemiology* 2018; **187**: 1530–8.

3 Gasperoni F, Jackson CH, Wood AM, *et al.* Optimal risk-assessment scheduling for primary prevention of cardiovascular disease. 2023; published online Feb 9. DOI:10.48550/arXiv.2302.04992.

4 Hippisley-Cox J, Coupland C, Vinogradova Y, Robson J, May M, Brindle P. Derivation and validation of QRISK, a new cardiovascular disease risk score for the United Kingdom: prospective open cohort study. *BMJ* 2007; **335**: 136–136.

5 Hippisley-Cox J, Coupland C, Vinogradova Y, *et al.* Predicting cardiovascular risk in England and Wales: prospective derivation and validation of QRISK2. *BMJ* 2008; **336**: 1475–82.

6 Hippisley-Cox J, Coupland C, Brindle P. Development and validation of QRISK3 risk prediction algorithms to estimate future risk of cardiovascular disease: prospective cohort study. *BMJ* 2017; **357**. DOI:10.1136/BMJ.J2099.

7 Townsend P, Phillimore P, Beattie A. Health and Deprivation: Inequality and the North. Croom Helm, 1988.

8 Herrett E, Shah AD, Boggon R, *et al.* Completeness and diagnostic validity of recording acute myocardial infarction events in primary care, hospital care, disease registry, and national mortality records: cohort study. *BMJ* 2013; **346**: f2350.

9 Xu Z, Arnold M, Stevens D, *et al.* Prediction of Cardiovascular Disease Risk Accounting for Future Initiation of Statin Treatment. *American journal of epidemiology* 2021; **190**: 2000–14.

10 Population estimates for the UK, England and Wales, Scotland and Northern Ireland - Office for National Statistics. 2020. https://www.ons.gov.uk/peoplepopulationandcommunity/populationandmigration/populationestimates/bulletins/annualmidyearpopulationestimates/mid2020#the-uk-population-at-mid-2020 (accessed Feb 23, 2023).
